# Bacteria-Human Somatic Cell Lateral Gene Transfer Is Enriched in Cancer Samples

**DOI:** 10.1371/journal.pcbi.1003107

**Published:** 2013-06-20

**Authors:** David R. Riley, Karsten B. Sieber, Kelly M. Robinson, James Robert White, Ashwinkumar Ganesan, Syrus Nourbakhsh, Julie C. Dunning Hotopp

**Affiliations:** 1 Institute for Genome Sciences, University of Maryland School of Medicine, Baltimore, Maryland, United States of America; 2 Computer Science and Electrical Engineering Department, University of Maryland Baltimore County, Baltimore, Maryland, United States of America; 3 University of Maryland College Park, College Park, Maryland, United States of America; 4 Department of Microbiology and Immunology, University of Maryland School of Medicine, Baltimore, Maryland, United States of America; 5 Greenebaum Cancer Center, University of Maryland School of Medicine, Baltimore, Maryland, United States of America; University of California Davis, United States of America

## Abstract

There are 10× more bacterial cells in our bodies from the microbiome than human cells. Viral DNA is known to integrate in the human genome, but the integration of bacterial DNA has not been described. Using publicly available sequence data from the human genome project, the 1000 Genomes Project, and The Cancer Genome Atlas (TCGA), we examined bacterial DNA integration into the human somatic genome. Here we present evidence that bacterial DNA integrates into the human somatic genome through an RNA intermediate, and that such integrations are detected more frequently in (a) tumors than normal samples, (b) RNA than DNA samples, and (c) the mitochondrial genome than the nuclear genome. Hundreds of thousands of paired reads support random integration of *Acinetobacter-*like DNA in the human mitochondrial genome in acute myeloid leukemia samples. Numerous read pairs across multiple stomach adenocarcinoma samples support specific integration of *Pseudomonas-*like DNA in the 5′-UTR and 3′-UTR of four proto-oncogenes that are up-regulated in their transcription, consistent with conversion to an oncogene. These data support our hypothesis that bacterial integrations occur in the human somatic genome and may play a role in carcinogenesis. We anticipate that the application of our approach to additional cancer genome projects will lead to the more frequent detection of bacterial DNA integrations in tumors that are in close proximity to the human microbiome.

## Author Summary

There are 10× more bacterial cells in the human body than there are human cells that are part of the human microbiome. Many of those bacteria are in constant, intimate contact with human cells. We sought to establish if bacterial cells insert their own DNA into the human genome. Such random mutations could cause disease in the same manner that mutagens like UV rays from the sun or chemicals in cigarettes induce mutations. We detected the integration of bacterial DNA in the human genome more readily in tumors than normal samples. In particular, extensive amounts of DNA with similarity to *Acinetobacter* DNA were fused to human mitochondrial DNA in acute myeloid leukemia samples. We also identified specific integrations of DNA with similarity to *Pseudomonas* DNA near the untranslated regulatory regions of four proto-oncogenes. This supports our hypothesis that bacterial integrations occur in the human somatic genome that may potentially play a role in carcinogenesis. Further study in this area may provide new avenues for cancer prevention.

## Introduction

Lateral gene transfer (LGT) is the transmission of genetic material by means other than direct vertical transmission from progenitors to their offspring, and has been best studied for its ability to transfer novel genotypes between species. LGT occurs most frequently between organisms that are in close physical proximity to one another [1]. Human somatic cells are exposed to a vast microbiome that includes ∼10^14^ bacterial cells that outnumber human cells 10∶1 [2]. Considering that (a) some human cells are in a constant and intimate relationship with the microbiome, (b) eukaryotes have widespread LGT from bacteria [3], (c) bacteria *in vitro* can transform the mammalian genome [4], and (d) viruses integrate into the human genome and cause disease [5], [6], we sought to investigate if LGT from bacteria to human somatic cells may be a novel mutagen and play a role in diseases associated with DNA damage like cancer.

Previous studies have examined LGT from bacteria to humans that would result in vertical inheritance. During the original sequencing and analysis of the human genome, 113 proteins putatively arising from bacterial LGT were initially identified [7]. This was later refuted by an analysis that demonstrated that the number of putative LGTs is dependent on the number of reference genomes used in the analysis suggesting that the proteins found exclusively in both bacteria and humans at that time were due to the small sample size of genomes sequenced, instead of LGT [8]. A subsequent phylogenetic analysis of LGT in the human genome overlooked comparisons with all prokaryotes [9]. Both analyses only focused on full length genes, missing any smaller LGTs or LGT of non-coding DNA. In addition, by focusing on consensus genome sequences, these analyses focused on LGT to the germ line and ignored somatic cell mutations. While LGT to the germ line can affect future generations and potentially the evolution of our species, LGT to somatic cells has the potential to affect an individual as a unique feature of their personal genome.

Some eukaryotes have extensive vertically inherited LGT despite potential barriers such as the nucleus, the immune system, and protected germ cells. DNA continues to be transferred from mitochondria and chloroplasts into the eukaryotic nucleus. These organelles originated from α-proteobacteria and cyanobacteria, respectively [10]. LGT from bacteria to eukaryotes, including animals, is also quite widespread [11]–[13], particularly from endosymbionts [3]. *Wolbachia* endosymbionts infect up to 70% of all insects [14], with ∼70% of examined, available invertebrate host genomes containing gene transfers [15]. The amount of genetic material transferred ranges from 100 bp [15], [16] to bacterial genome sized LGTs [15], [17], [18].

One of the best studied examples of LGT from bacteria to eukaryotes is LGT to plants from the bacteria *Agrobacterium tumefaciens*. *A. tumefaciens* uses a type IV secretion system to inject bacterial proteins and its tumor inducing plasmid into plant cells [19]. Through illegitimate recombination, the plasmid integrates into the plant genome, and plasmid encoded transcripts are produced using endogenous eukaryotic promoters [20], [21]. The corresponding proteins create a specific carbon source for *A. tumefaciens* and promote the formation of plant tumors [19], [22]. Therefore, *A. tumefaciens* creates a tumor environment that promotes the bacteria's own growth. *A. tumefaciens* has been shown to transform a variety of plant and non-plant cells including human cells *in vitro* [22], [23].

The bacteria *Bartonella henselae* has also been shown to transform human cells *in vitro. Bartonella henselae* is a human opportunistic pathogen that causes cat-scratch disease [24]. *B. henselae* and *B. quintana* are the only known bacteria to cause bacillary angiomatosis, the formation of benign tumors in blood vessels [24], [25]. A recent study demonstrated the ability of *Bartonella henselae* to integrate its plasmid into human cells *in vitro* through its type IV secretion system [26].

Bacterial plasmids have also been engineered to integrate autonomously in vertebrate genomes using the phiC31 integrase. A phiC31 integrase-containing plasmid was first shown to integrate into human cells *in vitro* [27] at a pseudo*-attP* site that does not disrupt normal gene functions. The plasmid also integrates into mice *in vivo* after hydrodynamic tail-vein injection [28] and can yield a properly expressed protein that rescues a mouse knockout phenotype [28].

One of the key mechanisms by which some viruses promote carcinogenesis is through their integration into the human genome, causing somatic mutations [29]–[31]. In the early 20^th^ century viruses were suggested as a transmissible cause of cancer. However, it was not until the mid-1960s that the capability of viruses to promote human cancer was fully recognized [29]. The majority of viral-associated human cancers are related to infection with human papillomaviruses (HPV), hepatitis B and C viruses, and Epstein-Barr virus. Together these viruses are associated with ∼11% of the global cancer burden [32]. In 2002, cervical cancers resulted in ∼275,000 deaths, of which HPV had integrated into ∼90% of these cancers [33].

Almost all cancers associated with Hepatitis B virus (HBV) have the virus integrated into tumor cells [34]. Most of the observed HBV integrations have been isolated as a single occurrence from a single patient [6]. However, a few recurrent integrations into genes promoting tumor formation have been identified, such as the integration of HBV into the human telomerase reverse transcriptase gene [35], [36]. These mutations can result in altered gene expression and promote carcinogenesis. The advent of next generation sequencing has facilitated the investigation of how and where these viruses integrate into the human genome with unprecedented resolution and accuracy. In a recent study, next generation DNA and RNA sequencing identified HBV integrations in liver cancer genomes and concluded that the HBV integrations disrupted chromosomal stability and gene regulation, which was correlated with overall shortened survival of individuals [6].

Using publicly available sequence data from the human genome project, the 1000 Genomes Project, and The Cancer Genome Atlas (TCGA), we examined bacterial DNA integration into the human somatic genome, particularly tumor genomes. Here we show that bacterial DNA integrates in human somatic genomes more frequently in tumors than normal samples. These data also support our hypothesis that bacterial integrations occur in the human somatic genome and may lead to altered gene expression.

## Results

### Identifying bacterial integrations in the somatic human genome

Human DNA for genome sequencing is typically isolated from one of three sources: sperm, blood, or cell lines created by transforming collected cells. Most of the data presented here from the Trace Archive and 1000 Genomes project were collected from the latter two. Systematic comparisons of the integration rate based on tissue source is not possible because the metadata on source can be missing, internally inconsistent, or at odds with publications of the data. However, it is important to consider that some of the data arises from cell lines. Cell lines may be more permissive to LGT from bacteria. Cell lines are used frequently because once they are generated they can be maintained in the laboratory allowing greater access to materials by more researchers. On the other end of the spectrum, transfers of bacterial DNA in sperm cells could be inherited by a subsequent generation. In contrast, transfers in blood cells would generate somatic mutations that would not be inherited. In addition, if a transfer occurs in a terminally differentiated cell its fate within the individual would even be limited.

**Figure 1:**
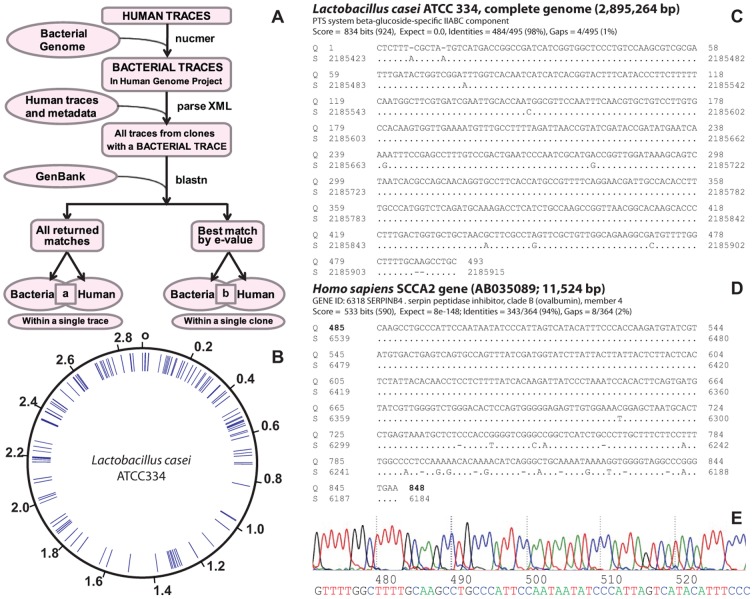
LGT from bacteria to human somatic cells using Trace Archive data. The schematic illustrates our pipeline that identified 319 clones (a) and 680 traces (b) with the hallmarks of LGT from bacteria to humans using Trace Archive data (Panel A). The traces and clones with similarity to *Lactobacillus casei* are randomly distributed across the bacterial genome (Panel B). The BLAST search results for one of these reads shows the left portion with similarity to *Lactobacillus casei* ATCC334 (Panel C), while the right portion of the read has similarity to the human SCCA2 gene (Panel D). The transfer of *Lactobacillus casei* DNA occurs in the fourth intron of the SCCA2 (SerpinB4) gene. The chromatogram (Panel E) shows the junction between the sequences in C and D and appears to be a single, high quality sequence trace.

Somatic mutations are frequently overlooked in genome sequencing as there may be only a single instance within the sequenced population of cells that is lost in the consensus-built genome assembly. Therefore, we examined all available human sequence traces for evidence of LGT to somatic cells. Previously, we had developed a pipeline for rapidly identifying LGT between *Wolbachia* and its hosts by using NUCMER [37] (Figure 1A). BLASTN against NT was used to further validate such transfers. Using this pipeline, 8 of the 11 hosts of *Wolbachia* endosymbionts that were examined were found to have evidence of LGT between the endosymbiont genome and the host chromosome [15]. In five of these hosts, we were able to successfully characterize every LGT we attempted to validate using standard laboratory techniques [15]. The other three hosts were not examined further.

### Bacterial LGT in the trace archive

Given our prior success with the NUCMER-based pipeline, we used it to search for LGT in the somatic cells of humans. We searched 113,046,604 human shotgun Sanger traces from 13 sequencing centers and >8 individuals with 2,241 bacterial genomes using NUCMER (Figure 1A). All reads were subsequently searched against NT with BLASTN (Figure 1A) and manually curated to identify (a) reads containing non-overlapping matches to human and bacteria sequences (Table S1) and (b) read pairs where one read matched human and the other matched bacteria (Table S2).

These searches revealed a total of 680 traces that contain significant non-overlapping similarity to both bacteria and human sequences (Figure 1Aa, Table S1). There are also 319 identified clones that contain sequences with similarity to both bacteria and human sequences (Figure 1Ab, Table S2). For example, 40 traces and 220 clones contain bacterial fragments with best blast matches to *Lactobacillus* spp. when NT was the database. These matches were found to be distributed across an entire *Lactobacillus* genome (Figure 1B) and could not be assembled. The lack of coverage/redundancy across the LGT junctions may be indicative of somatic cell transfers. As an example, one such trace is illustrated that disrupts a gene encoding an antigen found in squamous cell carcinomas [38] (Figure 1CD). The trace containing this junction does not show evidence of an artifact (e.g. two clones being sequenced simultaneously) (Figure 1E).

Laboratory artifacts can lead to sequences resembling bacteria-eukaryote somatic cell LGT. Errors can occur in clone or sequence tracking, such that traces are assigned to the wrong project, or through contamination of plasmid preparations that leads to two sequences being generated simultaneously. Some cases of these were identified and systematically culled. For example, reads with matches to *E. coli* were systematically eliminated because of the high potential for artifactual contamination of genomic DNA in plasmid sequencing preparations. Similarly, all matches involving *Erythrobacter* were eliminated since a set of traces submitted by one center were found to contain two sequences—one for human and one for *Erythrobacter* likely owing to systematic contamination of the culture stocks or the plasmid preparations. When two templates are present the resulting read will switch between the two templates as the relative signal between the templates changes resulting in a consensus read call that resembles LGT. However, such artifacts are not readily apparent for any of the putative LGTs described her since the sequences span multiple plates, libraries, and runs and show no evidence of two templates (Table S1, S2).

Ligation of bacterial DNA to human genomic DNA during library construction can also result in chimeric clones with a single clone with a bacterial insert and a human insert. This would be observed as a low percentage of bacteria-human mate pairs relative to bacteria-bacteria mate pairs. For example, if 1 in every 100,000 clones contains two inserts, as opposed to the single insert wanted/expected, one would expect a chimeric clone with both a human and bacterial insert would occur no more than 1/100,000, or 0.001%. Considering that human sequences greatly outnumber bacterial sequences, we would expect clones with bacteria and human inserts to occur much less frequently than human-human chimeras and that the number of bacteria-human chimeras will be almost solely based on the amount of bacterial DNA in the samples. We would also anticipate that if 0.001% of bacterial reads are found in bacteria-human chimeric clones then 0.001% of human reads will be found in human-human chimeric clones and be discordant in the human genome.

However, we find that the percentage of reads or read pairs supporting integration relative to the number of human mate pairs is higher than one would anticipate or has been measured previously. The average percentage of bacteria-human mate pairs compared to bacteria-bacteria mate pairs is ∼6% (319 highly curated bacteria-human clones/5,280 minimally curated bacteria-bacteria clones), meaning 6% of the bacteria sequences are attached to human sequences. If the bacteria-human sequences were the result of artifactual chimeras, we would expect that 6% of the human sequences should also be erroneously attached to non-adjacent human sequences. This level of artifact chimerism would undermine assembly as well as results regarding human genome structural variation. To the contrary, one such structural variation study found that <1% of the mate pairs were discordant with the reference human genome [39] using some of the same genome sequencing data used here. While it would be prudent to measure the human-human chimerism rates across all the data to compare to the bacteria-human chimerism rates, the lack of a strict ontology for the metadata precludes this. Specifically, it is difficult to determine the exact nature of the pertinent data needed (i.e. sequencing strategy and insert size) for such an analysis.

### Identifying LGT in next generation sequencing data

**Figure 2:**
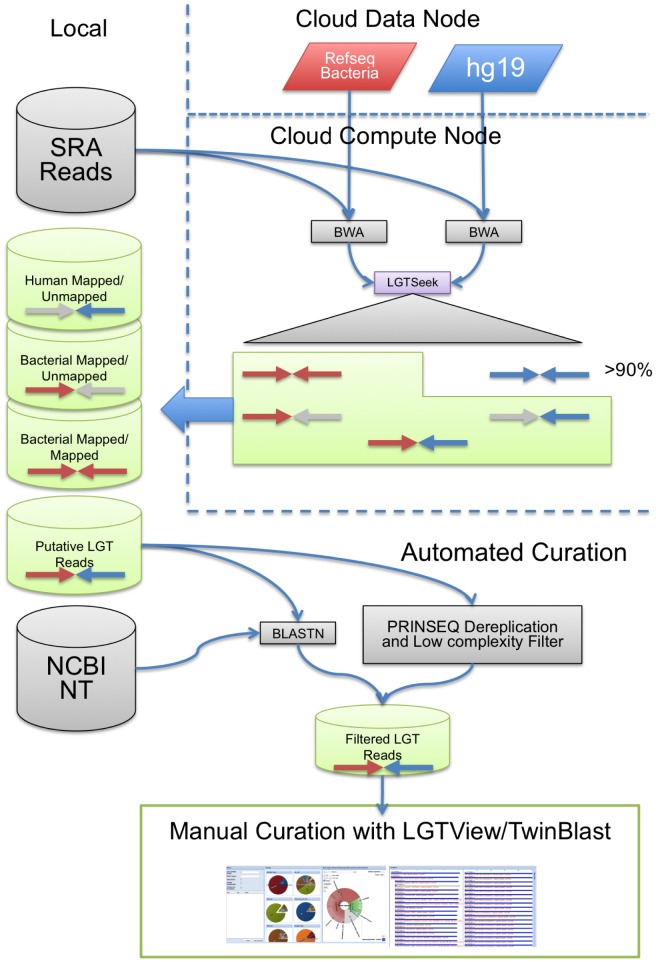
Cloud-based method for identifying putative LGT reads. Sequencing files containing paired-end sequences were uploaded to a CloVR virtual machine on the DIAG. Complete bacterial genomes from RefSeq and the human genome reference hg19 were downloaded from a persistent data node in the DIAG. The sequencing queries were mapped to the two references using BWA. The mappings were processed using LGTSeek, which classifies reads based on their mapping profiles. All mappings except for human/human were downloaded to local storage at the completion of the analysis. Next, putative LGT reads were run through automated curation steps including a BLAST search against NT and PrinSEQ dereplication to remove PCR duplicates and low complexity filtering (Figure S1). These filtered reads were then loaded into a database and inspected manually through a custom graphical interface.

In order to extend this observation to next generation sequencing data, we created a pipeline (Figure 2, Figure S1) to identify Illumina paired end reads that consist of one bacterial read and one human read in the 1000 genomes and TCGA datasets. This is analogous to identifying bacteria-human mate pairs with NUCMER above (Figure 1A, left side). The first round of filtering uses BWA [40] to map the paired end reads to the human reference and the completed bacterial genomes in the RefSeq database. BWA was run with the default parameters such that the number of differences is dependent on the read length; for example a 50-bp read has 3 differences allowed [40]. BWA was designed to align short query sequences against much longer reference genomes with great efficiency. It was chosen as the initial screen because it could efficiently process very large datasets quickly. After BWA identified a small subset of the paired end reads that support bacterial integration, BLASTN was used to validate each read of the pair as specific for bacteria or human using the larger NT database. Subsequently, a lowest common ancestor (LCA) approach [41] was used to assign operational taxonomic units (OTUs) to each read using either the best BLASTN matches to NT or all of the results of BWA searches against the completed bacterial genomes in RefSeq. As expected, the level of taxonomic assignment possible was largely dictated by the sequence variation in the reference sequences used, as seen with a comparison of sequences with similarity to the 16S rRNA gene and what is known about the variable and conserved regions of that gene. (Figure S2). The results of BWA-based and BLAST-based LCA assignment methods each have their nuances but the results were very similar and parsimonious with a phylogenetic analysis (Figure S3). Problems were identified with using BLAST searches against NT due to eukaryotic whole genome sequencing projects that likely contain contigs from the microbiome (Figure S3). As such, the BWA-based LCAs are presented here. Regardless, even when specific (e.g. strain level assignments) OTUs should never be deemed definitive and should merely be considered an approximation of the taxonomy of the sequence. The blast-based assignments and subsequent analysis is available in tables and in an interactive interface for the 1000 genomes data (Table S3; http://lgt.igs.umaryland.edu/1000genomes) and TCGA data (Table S4; http://lgt.igs.umaryland.edu/tcga).

**Figure 3:**
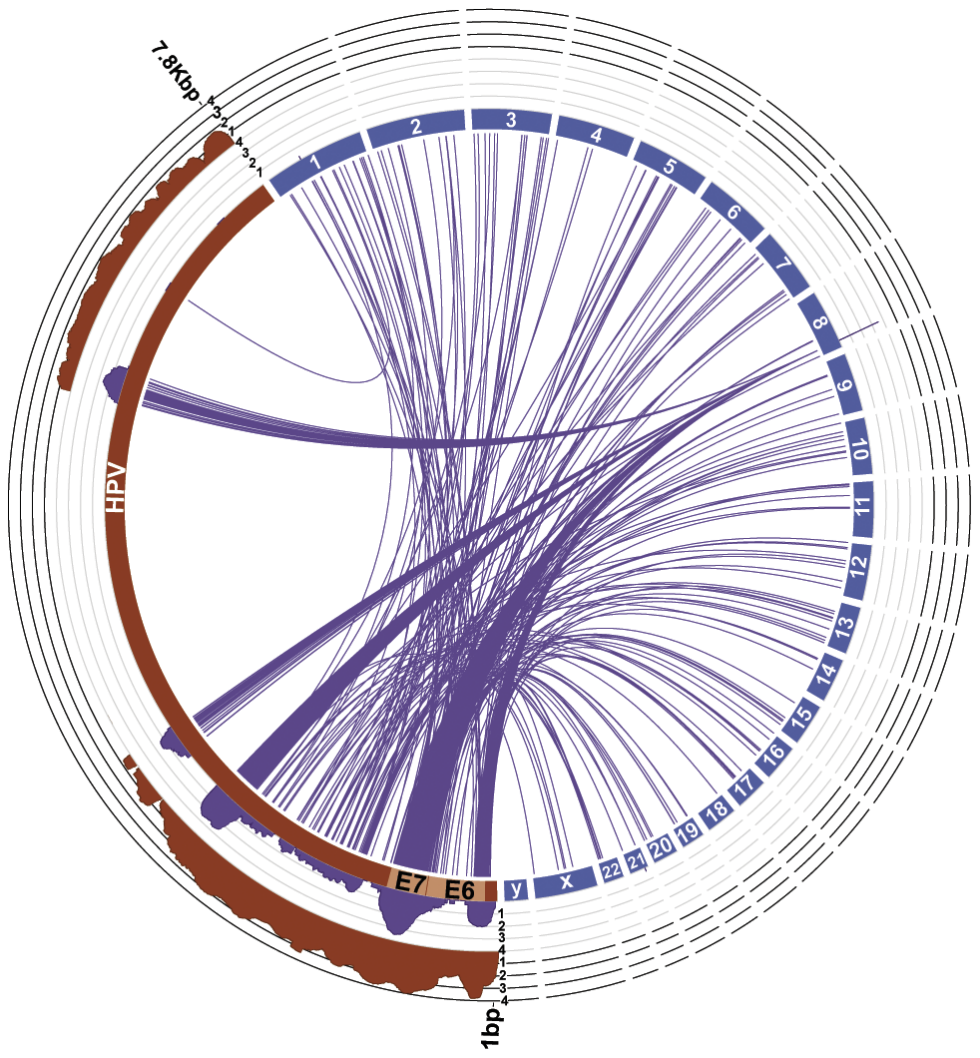
Identification of HPV Integration into the HeLA genome. As a control, integrations of the human papillomavirus genome NC_001357 (red) into the HeLa cell genome represented by hg19 (blue) were detected with our pipeline. The integration of HPV into chromosome 8 in the HeLa genome is supported by read pairs with one read mapping to HPV and the other mapping to the human genome (purple lines). The log-transformed coverage of the reads supporting integration (purple histogram, axis minimum = 0, axis maximum = 4) is consistent with the known integration of the HPV E6 and E7 genes shown in pink on the HPV genome. The log-transformed coverage of the viral mate pairs is also shown (red histogram).

To calibrate our pipeline, we reconstructed the known HPV integration in HeLa cells using available RNA-based Illumina sequence data [42]. The HeLa cell line has a well-documented integration of HPV into chromosome 8 as well as constitutive expression of the viral oncogenes E6 and E7 [31], [42]–[44]. Previously, PathSeq was used to identify 25,879 HPV reads in the HeLa transcriptome (0.25% of the total reads analyzed) [42]. Using the same transcriptomics data, our pipeline identified a similar number of 28,368 paired-end reads (0.55% of the total read pairs) with both reads mapping to HPV. Furthermore, our pipeline identified 6,333 reads (0.12% of the total read pairs) supporting integration of HPV into the human genome. These paired end reads span the viral integration site, with one read mapping to HPV and the other read mapping to the human genome (Figure 3). As expected, the reads supporting the HPV integration into the human genome flanked the constitutively expressed E6 and E7 viral oncogenes. The human portions of these paired end reads reside in the known tandem HPV integration site on chromosome 8 between 128,240,832–128,241,553 bp [33], [45], [46].

### LGT in the 1000 Genomes Project

Using this pipeline on 3.15 billion Illumina read pairs from the 1000 Genomes Project available as of February 2011, 7,191 read pairs supported bacterial integration into the somatic human genome after BLASTN validation, removal of PCR duplicates, and a low complexity filter. The integrations have up to 5× coverage on the human genome. Of the 484 individuals examined, 153 individuals have evidence of LGT from bacteria with 1 individual having >1000 human-bacteria mate pairs and 22 individuals having >100 such pairs. On average, 47 human-bacteria mate pairs were identified in these individuals with putative somatic LGT (median = 2; maximum = 1360). These putative somatic cell LGTs were identified in data from all five centers that contributed data to this release.

*Bradyrhizobium* was the most common OTU identified in the reads supporting LGT, with *Bradyrhizobium* sp. BTAi1 being the most common strain-level OTU. The *Bradyrhizobium*-like reads were distributed across an entire reference *Bradyrhizobium* genome (Figure S4A) similar to what was observed for *Lactobacillus* sequences in the Trace Archive data (Figure 1B). BTAi1 is a strain that is unusual in its ability to fix nitrogen and carry out photosynthesis. Therefore, some may consider the presence of BTAi1-like sequences in humans unusual. However, our understanding of what bacteria exist in the body is limited. Most of the samples containing *Bradyrhizobium-*like reads were from the Han Chinese South (CHS) population and were sequenced by the Beijing Genomics Institute (BGI). OTUs associated with bacterial integration that were detected in only one center may be viewed suspiciously, and several, including this one, were observed. However, population level differences in the diet, life style, and microbiome of the different populations examined could also lead to this result. The CHS study is an example of the difficulties in ascertaining the source of the material sequenced. The study information in the SRA states that lymphoblastoid cell lines were used (SRP001293; http://trace.ncbi.nlm.nih.gov/Traces/sra/sra.cgi?study=SRP001293), but the sample information states that blood was used (http://trace.ncbi.nlm.nih.gov/Traces/sra/sra.cgi?view=samples).

Two OTUs—*Propionibacter acnes* and Enterobacteriaceae—were detected in samples from all five centers. *P. acnes* is a common skin bacteria that is associated with acne. It is thought to contaminate genomic DNA preparations either from laboratory workers or during sample collection. Whether bacterial DNA arises from contaminants or the microbiome, laboratory artifact chimeras in Illumina whole genome shotgun sequencing that resemble bacterial integrations can occur (a) during PCR amplification steps in library construction or (b) from over-clustering on the flow cell [47]. The other OTU found across all five centers is a family level assignment of Enterobacteriaceae, which includes *Escherichia coli*. While next generation sequencing no longer relies on plasmid-based clones, they do use ligation steps and recombinant enzymes isolated from *E. coli*. Therefore, it is quite possible that low levels of *E. coli* DNA could be introduced with the enzyme preparations. Because both *E. coli* and *P. acnes* DNA in samples could arise from contamination of the samples, out of an abundance of caution they were excluded from all analyses. However, we note that this may be a conservative approach given that other Enterobacteriaceae may be found in the samples besides *E. coli* and both *E. coli* and *P. acnes* could contribute to bacterial integration.

### Distinguishing bacterial integrations from laboratory artifacts

Given that the putative LGTs detected are likely some combination of real LGT and laboratory-based artifacts of reads from the microbiome, we sought to establish a metric by which the two could be differentiated. Given the short length of these reads, our analysis of next generation sequencing data focused solely on Illumina paired end data, identifying putative bacterial integrations when one read mapped to human and one to bacteria. Due to the length of the reads, chimeric reads could not be identified with BWA (e.g. a 50-bp read that had 25-bp mapping to a bacteria and 25-bp mapping to human could not be identified with BWA because it would remain unmapped). Given the sole use of paired end data, reads from the microbiome were defined as those where both reads only map to a bacterial genome. This is, however, an oversimplification since any integration of bacterial DNA larger than the library insert size is likely to generate such reads. Regardless, the microbes that contribute to putative LGT are just a subset of the microbes present (Figure S5). If junctions of bacteria-human read pairs are merely artifacts, one would anticipate that they form in the same proportion relative to the contaminating DNA. However, this was not observed (Figure S5).

**Figure 4:**
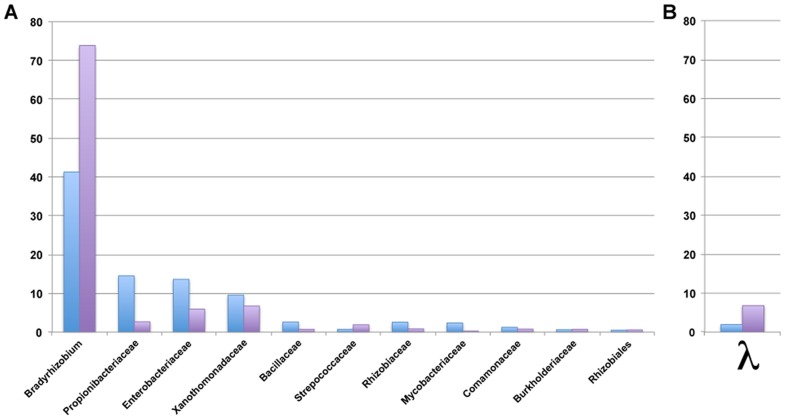
Relative proportion of OTUs in the microbiome compared to the proportion in bacterial DNA integration. The relative contribution of an OTU at the family level is shown (Panel A) for the microbiome (blue) and bacterial integrations (purple). OTUs that are over-represented in the microbiome include several common lab contaminants that were observed at low levels across multiple samples and centers (e.g. *P. acnes*). OTUs that are over-represented in the bacterial integrations are more likely to be the organisms mutagenizing the human somatic genome. The contribution of λ phage microbiome (blue) and integrations (red) was measured and illustrated separately (Panel B).

Each OTU could be binned into one of two categories based on the difference between the composition of the microbiome and the LGT reads: (A) one where the contribution of the specific bacteria relative to the total population of bacteria is higher in the reads supporting LGT and (B) one where the contribution of the specific bacteria relative to the total population is higher in the reads coming from the microbiome. One would anticipate that the former would contain bacteria participating in real LGT, since the proportion of reads with putative LGT is higher while the latter would represent the level of artifactual chimeras from contaminating DNA. This cannot be examined on a per sample basis since most samples have a limited amount of bacterial DNA. However, when the data is aggregated across the entire project (Figure 4A), the bacteria do in fact fall into either of these two categories. As expected, bacteria in the families of Propionibacterineae and Enterobacteriaceae fall into category B, along with Xanthomonadaceae. In contrast, Bradyrhizobiaceae falls into category A.

In a preliminary analysis, the phage λ was observed to fit into category A. In the above analysis, it is not observed because λ, a bacteriophage, has similarity to sequences with an NCBI taxonomy of “cloning” and “expression vector” that are excluded with our final criteria. However, if we specifically include the λ reads, λ falls within category A (Figure 4B). The reads map only to a small portion of the λ phage, specifically ranging in coverage from 50×–250× on both sides of a HindIII site. It is possible that this is a contaminant as λ is commonly used in research labs. For instance, an excised gel slice may have been contaminated with a λ fragment from an adjacent lane containing a λ ladder. However, this is not consistent with having reads on both sides of the same HindIII site. If the slice was contaminated with two ladder fragments, we would anticipate equal numbers of reads at two additional sites reflecting both ends of the fragment, which was not observed. We could not reconstruct, with *in silico* digestions of common and uncommon restriction endonucleases, a scenario that explains our observation and reflects what is known about laboratory artifacts in genome sequence data. Should this integration of λ in the human genome be validated, it is intriguing since a phiC31 integrase-containing plasmid has already been shown to integrate into human cells *in vitro* [27] at a pseudo*-attP* site. If prophage can integrate naturally into the human genome, they may also be capable of producing virions that would serve as an immune defense against certain bacteria.

### Rate of integration of bacterial DNA in the human genome

To further explore the relationship between bacterial integrations and laboratory artifacts, we sought to establish the mutation rate across each dataset as well as within subsets. The Trace Archive and 1000 Genomes data are derived from terminally differentiated blood samples, where integrations are expected to occur in a single generation. In the Trace Archive data [7], [45], [48] a total of 680 traces contain significant non-overlapping similarity to both bacteria and human sequences and 319 clones contain both bacteria and human sequences (Tables S1 and S2). From this data, an integration rate was measured as 680 integrations in 113,046,604 reads per a single generation, or 6.02×10^−6^ integrations/generation. While this may be considered an overestimate due to known laboratory artifact chimeras that result from cloning, it may also be an under-estimate as reads deposited in the Trace Archive are often cleansed of reads believed, but not proven, to be from bacterial contaminants. In the Illumina reads from the 1000 Genomes Project [49], 7,191 read pairs supporting integration were detected in 3,153,669,437 paired reads sequenced yielding a remarkably similar mutation rate of 2.28×10^−6^ integrations/generation assuming the mutations happen in a single generation.

This mutation rate would reflect both integrations as well as the formation of laboratory artifactual chimeras. To establish the contribution of the laboratory artifacts, we examined putative integrations involving OTUs of *Propionibacterium*. If reads with this OTU arose from contamination, then any bacteria-human read pairs would arise from laboratory artifacts. Of the 845,260,743 read pairs in runs containing putative integrations and/or reads attributed to the microbiome with a *Propionibacterium-*level OTUs, 191 read pairs represented putative integrations, yielding a mutation rate of 2.26×10^−7^, or 10-fold lower than that for the entire dataset. A similar analysis of λ, which may represent true integrations for the reasons outlined above, reveals 554 reads supporting integration out of 404,243,537 read pairs, or a mutation rate of 1.37×10^−6^, which is 6-fold higher than the *Propioinibacterium* rate.

### Coverage lends support for integrations

Coverage across a bacterial integration would provide greater evidence of its validity and would be observed when more than 1 unique read is present at a single site. Uniqueness of the reads was assessed with PRINSEQ after concatenating the two reads together and identifying if they are identical. Such identity of both sequence and insert size suggests that the pair are either PCR or optical duplicates formed during library construction or sequencing, respectively, and that should be counted only once. If coverage of unique read pairs supporting LGT across the human genome can be observed, it may suggest clonal expansion of a population with the LGT and support that they were formed biologically *in vivo* rather than through laboratory-based artifact formation *in vitro*. When the analysis is limited to putative LGT with >1× coverage on the human genome, only 275 read pairs support somatic cell LGT. The most predominant bacterial species level OTU, with 100 read pairs, is *Stenotrophomonas maltophilia*, an emerging opportunistic pathogen of the respiratory and blood systems of immunocompromised individuals [50]. The *Stenotrophomonas-*like reads were evenly distributed across the bacterial genomes (Figure S4B). Reads supporting *S. maltophilia*-like LGT were detected in two individuals in the study of Utah residents with Northern and Western European ancestry (CEU) sequenced at the Max Planck Institute of Molecular Genetics (MPIMG). One individual had the majority with 97 of these read pairs. While read pairs with >1× coverage were only detected in two samples from one site, when the coverage limit was relaxed, 450 read pairs with a *S. maltophilia* level OTU were detected in both the CEU and CHS studies and from both MPIMG and BGI.

While compelling, given the low coverage, the data from the 1000 Genomes Project is inconclusive in the absence of experimental validation. Yet, in terminally differentiated cells, like blood cells that are routinely sequenced, somatic cell LGT cannot be validated because the transfer sequenced was destroyed in the process of sequencing and is likely the only copy that exists. Transfers could occur in progenitor cells but as they are typically well protected, it is less likely. Furthermore, extensive coverage is not expected for the same reason. In several cases, we could identify coverage that further supports the validity of these reads but these instances were quite limited. In addition, much of the 1000 Genomes data examined are from the first pilot study that only generated 0.5–4× coverage of the genomes. Lastly, much of the DNA for the 1000 Genomes Project is derived from cell culture, not directly from blood cells. There is an opportunity for LGT to happen in cell culture that would not necessarily be biologically relevant. Therefore, we sought to validate these results further by examining data from cancer samples in TCGA.

### Analysis of TCGA data

**Figure 5:**
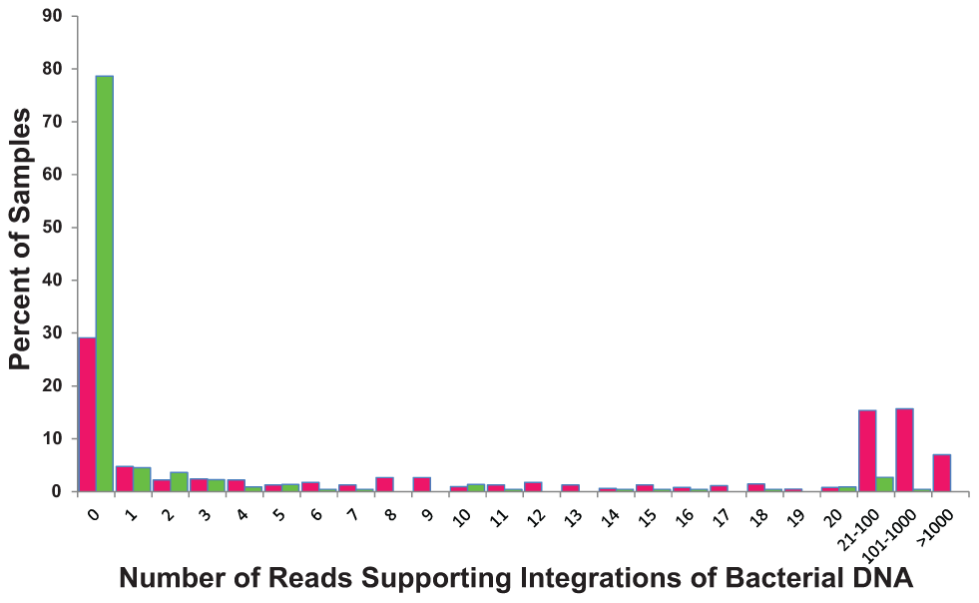
Distribution of reads supporting bacterial DNA integration into normal and cancer genomes. The percentage of samples is shown that contain a given number of paired reads that support integration of bacterial DNA in the tumor genomes (pink) and in the normal matched genomes (green).

From 7.05 trillion bases of Illumina paired-end sequencing data in TCGA, 691,561 read pairs support bacterial integration into the somatic human genome (Table 1). The integrations into the human genome have >100× sequencing coverage (Figure S6). TCGA contains sequencing data of tumor samples as well as normal tissue. Strikingly, while only 63.5% of TCGA samples analyzed were from tumors, the tumor samples contained 99.9% of reads supporting bacterial integration. Furthermore, while the majority of normal samples had no read pairs supporting integrations, the majority of tumor samples had >10 reads supporting integrations (Figure 5). However, these numbers may be biased by what was sequenced in each category (Table 1). For example, the two cases with extensive LGT lack matched normal samples and were both RNA-Seq studies.

**Table 1:** Summary of TCGA data analyzed by tumor type.

Cancer	Samples - C†	Samples -N†	Int. Reads - C†	Int. Reads - N†	Bac. Reads - C†	Bac. Reads - N†	Tot. Pairs - C†	Tot. Pairs - N†	Int. Freq - C†	Int. Freq. - N†	C/N‡
**LAML** *	130	0	665,676	ND*	29,633,118	ND	7,955,502,437	ND	8.4×10^−5^	ND	672
**BRCA** *	94	5	9,732	39	995,284	4,715	6,093,925,360	313,199,763	1.6×10^−6^	1.2×10^−7^	12.8
**GBM** *	69	70	118	122	11,857	11,357	5,391,069,119	5,295,464,216	2.2×10^−8^	2.3×10^−8^	1.0
**KIRC** *	79	0	1,906	ND	364,608	ND	5,070,366,679	ND	3.8×10^−7^	ND	3.0
**KIRP** *	9	0	175	ND	53,147	ND	541,775,677	ND	3.2×10^−7^	ND	2.6
**LUSC** *	20	0	995	ND	109,019	ND	1,166,304,426	ND	8.5×10^−7^	ND	6.9
**LUAD** *	14	0	191	ND	30,825	ND	980,790,987	ND	1.9×10^−7^	ND	1.6
**LIHC** *	0	1	ND	20	ND	6540	ND	66,799,150	ND	3.0×10^−7^	ND
**OV** *	146	144	722	852	13,161	25,087	8,643,898,191	7,716,679,202	8.4×10^−8^	1.1×10^−7^	0.8
**STAD** *	71	0	11,013	ND	4,860,934	ND	6,689,562,270	ND	1.6×10^−6^	ND	13.2
**TOTAL**	632	220	690,528	1,033	36,071,953	47,699	42,533,195,146	13,392,142,331	1.6×10^−5^	7.7×10^−8^	210

* LAML: Acute myeloid leukemia; BRCA: Breast invasive cancer; GBM: Glioblastoma multiforme; KIRC: Kidney renal clear cell carcinoma; KIRP: Kidney renal papillary cell carcinoma; LUSC: Lung squamous cell carcinoma; LUAD: Lung adenocarcinoma; LIHC: Liver hepatocellular carcinoma; OV: Ovarian serous cystadenocarcinoma; STAD: Stomach adenocarcinoma; ND = Not determined.

† Samples – C: Cancer samples; Samples – N: Normal samples; Int. Reads – C: Reads supporting integration from cancer samples; Int. Reads – N: Reads supporting integration from normal samples; Bac. Reads – N: Read pairs mapping to bacteria in cancer samples; Bac Reads – N: Read pairs mapping to bacteria in normal samples; Tot. Pairs – C: Total read pairs in cancer samples; Tot. Pairs – N: Total read pairs in normal samples; Int. Freq – C: Integration frequency in cancer samples; Int. Freq. – N: Integration frequency in normal samples.

‡ C/B is the integration rate in cancer samples divided by the integration rate in normal samples. When a normal was not available (LAML, KIRC, KIRP, LUSC, LUAD, STAD) the value from BRCA was used. BRCA was chosen because it is RNA sequencing as are the samples being examined that lack normal samples.

### Acute myeloid leukemia

Acute myeloid leukemia (LAML) was identified as the cancer type with the highest number of reads supporting integrations. This blood-derived cancer had 665,676 read pairs supporting putative integrations. Unfortunately, no normal samples were available in this data release for comparison. After identifying reads supporting bacterial integrations, we increased our stringency by requiring integrations to be supported by >4× unique coverage on the human genome. Implementing this criterion reduced the number of putative integrations to 90,726 paired end reads. When a genus level bacterial OTU could be identified, it was most frequently *Acinetobacter* with 31% of the reads (Figure S7). Moraxellaceae was the largest family level OTU (36%), which includes *Acinetobacter*. As with the 1000 Genomes data, a broader diversity of OTUs are observed in the microbiome reads than in the reads supporting LGT. The samples can be binned into one of five categories based on the microbiome (Figure S7C). Intriguingly, one of those categories lacks bacterial integration (Figure S7D, blue) and another has an extensive diversity of bacterial integrations across many OTUs (Figure S7D, dark green).

**Figure 6:**
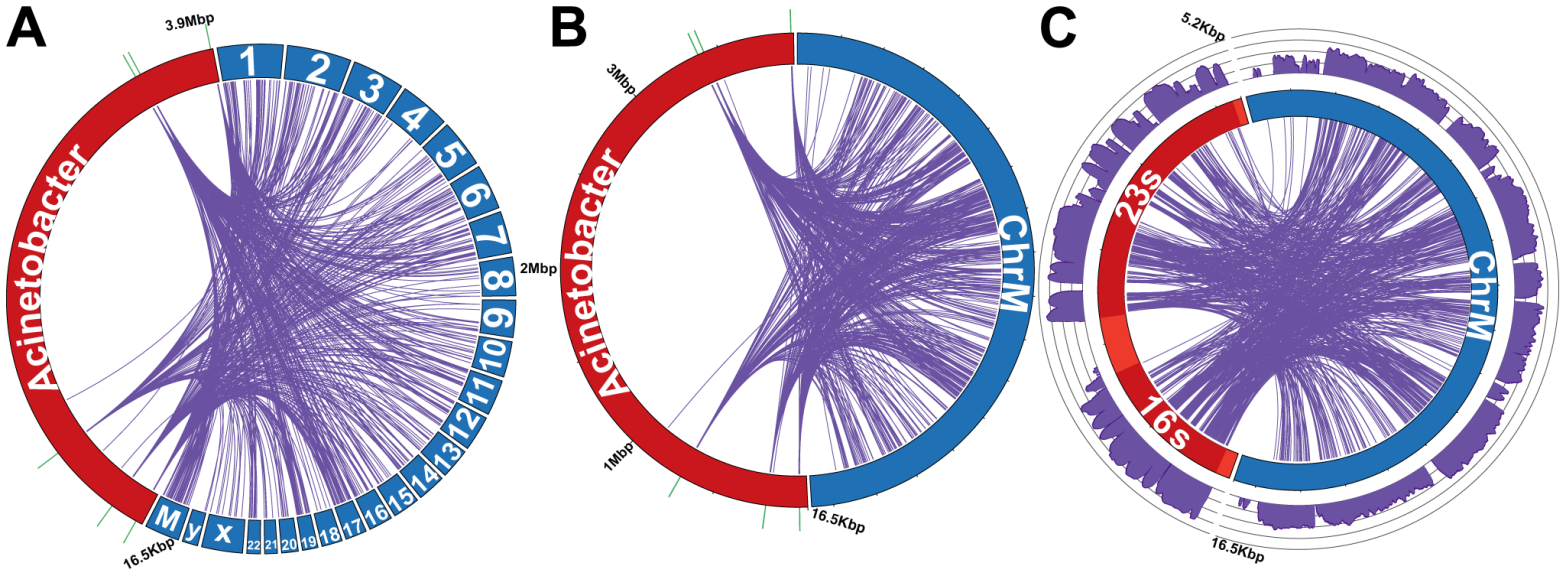
*Acinetobacter*-like integrations into the genome of acute myeloid leukemia samples. While putative integrations (purple lines) of *Acinetobacter*-like DNA (NC_010611.1) could be found in the nuclear genome, they were more abundant in the mitochondrial genome (Panel A, not drawn to scale). The integrations into the human mitochondria genome (blue) from an *Acinetobacter* spp. OTU in acute myeloid leukemia are mapped to the *Acinetobacter* genome (Panel B, red) or just the *Acinetobacter* rRNA (Panel C, red). The putative integrations into the mitochondria genome are randomly distributed across the entire genome while the bacterial sequences are mostly limited to sequences from the rRNA operon. The read coverage supporting integration is plotted on an ln-transformed scale (purple, axis minimum = 0, axis maximum = 5). The locations of the rRNA operons are denoted with green ticks on the outside rims of Panel A and B. Only integrations with an average of >4× coverage on the human genome are shown, and the data was down-sampled according to the methods.

Of the 90,726 reads supporting bacterial integrations into the human genome, 57,826 of those reads can map to the *Acinetobacter baumannii* genome (NC_010611) (Figure 6). Within the *Acinetobacter baumannii* genome, reads were frequently detected in the rRNA operon (57,487 reads in 5,279 bp) (Figure 6). Across the entire dataset, integration of rRNA was observed most frequently. For example, 68% of the reads attributed to the microbiome in LAML samples were from bacterial rRNA and 32% were from bacterial coding sequences (CDSs) (Table 2). Yet, 99% of the bacterial reads in LAML read pairs supporting bacterial integration were from rRNA and only 1% were from CDSs (Table 2).

**Table 2:** Genomic features from which LGT originates.

Feature	LAML microbiome		LAML LGT	
CDS	9,409,443	32%	749	0.87%
Gene	42	<0.01%	0	0%
Mature peptide	418	<0.01%	0	0%
Miscellaneous RNA	18,766	0.06%	0	0%
Noncoding RNA	2,797	0.01%	7	0.01%
Miscellaneous feature	20	<0.01%	0	0%
rRNA	19,843,921	68%	85,471	99%
Signal peptide	4,058	0.01%	1	<0.01%
tmRNA	0	0%	0	0%
tRNA	23,489	0.08%	57	0.07%
**Sum**	29,302,954	1	86285	1

In LAML, not only was there a preference for what bacterial DNA was integrated but also for the location of integration. Reads supporting bacterial DNA integration were detected more frequently in the human mitochondrial genome (Figure 6A, 41,852 reads in 16.6 kbp) than in the human nuclear genome (48,874 reads in 2.86 Gbp; p<2×10^−16^, Chi-squared test). The reads supporting integration were uniformly distributed across the entire mitochondrial genome with 10,085 unique read start sites (p = 0.27, thus rejecting the hypothesis that they are not random, Kolmogorov-Smirnov test, Figure 6BC). This is important because one might anticipate that bacterial rRNA preferentially integrates into the mitochondrial rRNA, but this was not observed. This also cannot be attributed to similarity between the *Acinetobacter* rRNA and the mitochondrial rRNA. There was no similarity detected between *Acinetobacter* rRNA and the human mitochondrial rRNA, or any other human sequence, as assessed by a BLASTN search of human genomic and transcriptomic sequences in NT with the Acinetobacter rRNA (data not shown). There also was no correlation observed between the amount of mitochondrial sequence in the sample and the number of integrants detected (Spearman rank coefficient p = 0.0681).

### Stomach adenocarcinoma

The stomach adenocarcinoma (STAD) cancer type had the second highest number of reads supporting bacterial integrations at 11,013. In the analysis of HBV integrations in human liver tumors, a criterion of a cluster of two read pairs was successfully applied to identify viral integrations in whole genome sequencing data with a validation success rate of 82% [6]. When a similar threshold requiring >1× coverage across the read (meaning at least two unique read pairs support the integration), the read count was still highest in LAML, followed by STAD, breast invasive cancer, and ovarian serous cancer. If the stringency is further increased, STAD samples contained 223 paired end reads with >4× coverage along the corresponding portion of the human genome. This high level of coverage lends great support for bacterial DNA integration. Unfortunately, STAD does not have normal matched samples for comparison in this data release.

**Figure 7:**
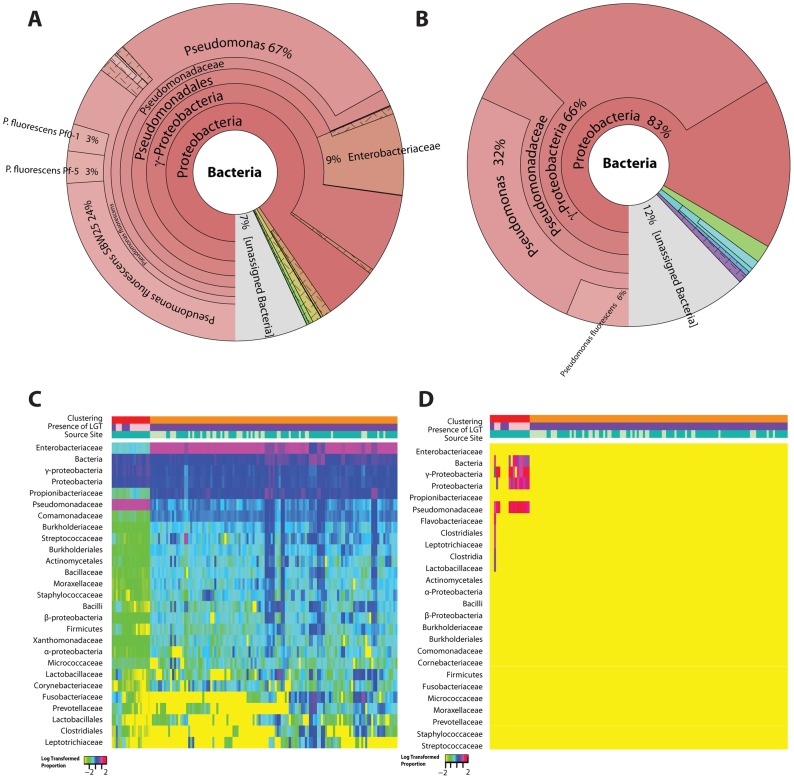
Distribution of bacterial OTUs from the microbiome and bacterial DNA integrations in stomach adenocarcinoma. The proportion of reads from each bacterial OTU is illustrated from the microbiome (Panel A) and LGT (Panel B) across all the stomach adenocarcinomas samples analyzed. The log-transformed proportion of the top bacterial OTUs per sample for the microbiome (Panel C) and LGT with >4× average coverage (Panel D) are clustered based on the microbiome profiles in Panel C and illustrated using heat maps. The relative proportion of taxonomic units related to *Pseudomonas* in the integrations was higher (as represented by red/hot pink) than in the microbiome (as represented by blue/purple).

The most common OTU (32%) for the bacterial integrations in STAD arose from the *Pseudomonas* spp. and related taxonomic units (Figure 7B). Approximately 6% of all reads supporting integration were more specifically assigned to the bacterial OTU *Pseudomonas fluorescens*. Of the 223 reads identified as bacterial integrations with >4× coverage on the human genome, 188 could be mapped to *P. fluorescens* (NC_012660). Of those, 184 mapped (98%) to the *P. fluorescens* rRNA operon (Figure S8) and only 4 integrations mapped to protein coding regions. *P. aeruginosa* has previously been shown to have a promoting effect on gastric tumorigenesis in rats receiving an alkylating agent [51]. Putative integration of DNA most likely of *Pseudomonas* origin has also been observed in the CBMI-Ral-Sto cell line in a study of NotI sites [52]; those *Pseudomonas*-like sequences have similarity to the ones we describe here (Figure S3J).

While *Helicobacter pylori* has been associated with the development of stomach cancer [53] only 2 Helicobacteraceae reads were identified supporting bacterial DNA integration, across all of the samples, and only 221 reads pairs with a Helicobacteraceae OTU were identified from the microbiome despite the presence of 15 *Helicobacter pylori* genomes in our reference dataset.

A clustering analysis of the microbiome reads separates the STAD tumor microbiome profiles into two clusters (Figure 7C). The tumors without integrations have a profile that is predominantly Enterobacteriaceae. However, the tumor samples with integrations have a distinct cluster of their own (Figure 7D) in which Pseudomonadaceae is the dominant OTU with a low proportion of Enterobacteriaceae. Although only one source site contributed to the sequencing of tumors with integrations, that same center also contributed samples of the other cluster. Furthermore, not all samples with a microbiome that is predominantly composed of members of the Pseudomonadaceae family had evidence of bacterial integration.

**Figure 8:**
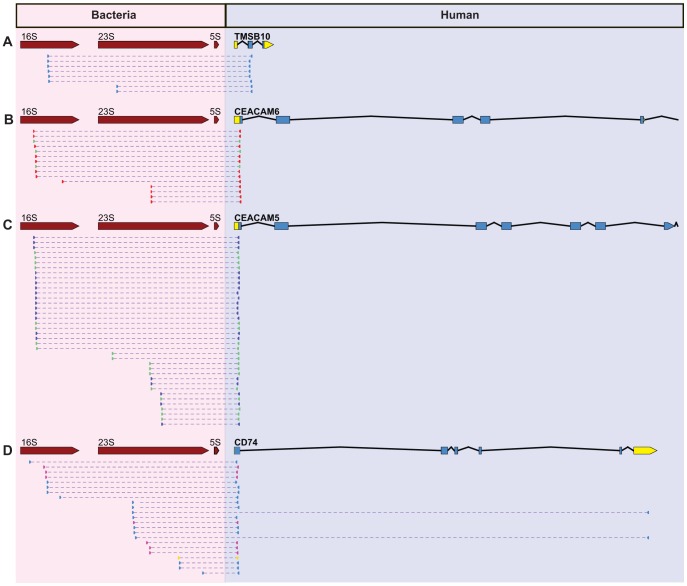
Integration sites of bacterial DNA in stomach adenocarcinomas. The read pairs supporting integration of DNA from a *Pseudomonas* OTU are illustrated between *Pseudomonas* rRNA operon (red) and the relevant portions of four human genes (blue) are shown for TMSB10 (Panel A), CEACAM6(Panel B), CEACAM5 (Panel C), and CD74 (Panel D). The human gene model with introns, exons, and untranslated regions (UTRs) are shown with the UTR highlighted in yellow. Putative integrations were frequently located near the 5′-UTR. Reads are color-coded by sample with multiple reads supporting each of these integrations and with some integration sites present in multiple individuals. Individuals are color-coded in the same manner in Figure 9. While the entire bacterial rRNA operon is shown, this is only for representative purposes. The transfer would include only a small portion that is relative to the library insert size, which is usually 300–400 bp for Illumina paired end data.

**Figure 9:**
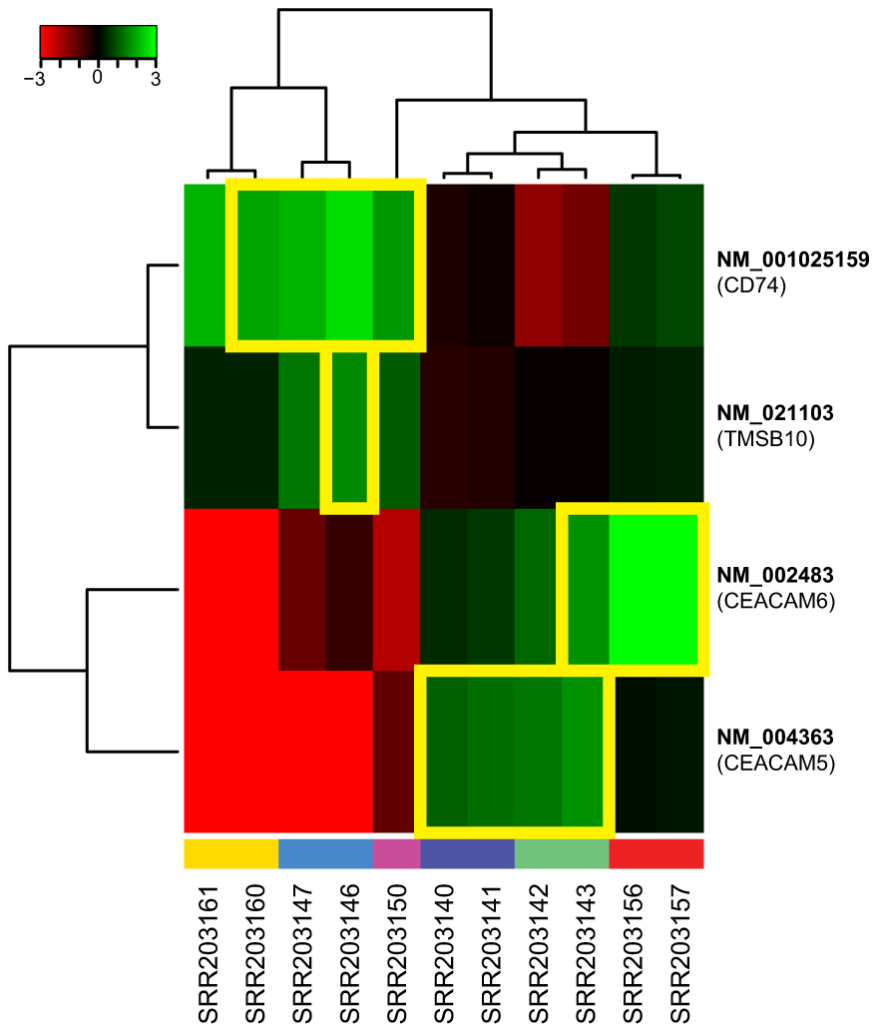
Differential expression analysis of transcripts associated with bacterial integrations in stomach adenocarcinomas. The log_2_(ratio) of the transcript abundance is illustrated for the ratio of the RPKM for the sample compared to the average RPKM across of the samples. Expression data for transcripts which have bacterial integrations are boxed in yellow. All of the transcripts with bacterial integrations are up-regulated relative to those that do not have such integrations. Individuals are color coded in the same manner as in Figure 8.

Most of these paired end reads supporting bacterial integration in the human genome were in five nuclear human genes (Figure 8A): TMSB10, IGKV4-1, CEACAM5, CEACAM6, and CD74. Four of these five integrations into STAD are in genes known to be up-regulated in gastric cancer, specifically CEACAM5, CEACAM6, TMSB, and CD74 [54]–[57]. An expression analysis reveals that genes with bacterial integration were all up-regulated relative to the average transcript level (Figure 9). Integration in genes up-regulated in stomach cancer (as opposed to those where transcription is down-regulated or abolished) is parsimonious with detecting integrations in tumor RNA, as we are unlikely to identify integrations that abolish transcription or transcript stability.

## Discussion

### Integration of bacterial DNA in the human somatic genome

Through this extensive analysis of several large human genome sequencing projects, we present evidence supporting LGT from bacteria to the human somatic genome. In terminally differentiated cells, we expect and observe that putative LGTs are detected consistently at low levels. Examination of clonally expanding tumors reveals many more transfers, as we would expect from a rapidly expanding population of cells. In all of the cases examined, the composition of the microbiome across the samples is different from the composition of bacterial DNA integrated into the human genome. When only the regions on the human genome with >4× coverage are examined, a pattern emerges of integration in the mitochondria for LAML and five genes in STAD. Remarkably, in STAD, four of those five genes have previously been shown to be implicated in cancer [54]–[57]. Together we believe this presents a compelling case that LGT occurs in the human somatic genome and that it could have an important role in human diseases associated with mutation.

While it is possible that these LGT mutations may play a role in carcinogenesis, it is also necessary to consider that they could simply be passenger mutations. The rapidly proliferating tumor cells may be more permissive to LGT from bacteria due to mutations in tumor suppressor genes or down regulation of DNA repair pathways. As a result of clonal expansion, rare mutations may be amplified throughout the tumor. Based on our analysis, it is impossible to determine if the LGTs have a causal role in cancer, or are simply a byproduct of carcinogenesis.

Likewise, while it is possible that the bacteria are causing mutations that benefit the bacteria, it is equally plausible that this occurs by random chance, or some combination of the two. If the mutations occur by random chance, mutations that induce carcinogenesis will be selected for over time within a local population of cells. This may explain why we observe low levels of LGT across the entire genome with increased coverage in specific genes in the STAD and LAML samples. In contrast, mutations that would benefit the bacteria would include those that create a micro-environment that promotes bacterial growth. This may explain why similar mutations, both in location and bacterial integrant, are observed in multiple individuals (Figure 8).

### Laboratory artifacts

While the extensive coverage across these putative integrations in multiple samples is strong support for bacterial integration being present in human tumors, we recognize the concern that such bacterial/human read pairs may arise merely from chimeric DNA generated during library construction. We pursued obtaining specimens for validation or establishing collaborations to accomplish this validation with TCGA investigators. Unfortunately, the combination of patient consent and access policy precludes the possibility of experimental validation on these samples by researchers that lack an IRB tied to a grant award from NCI/TCGA. As our funding is from the NIH New Innovator Program this was not possible. Collaborating with current TCGA investigators was also pursued but was found to be explicitly forbidden. However, we anticipate the future successful validation of these results by researchers with access to samples and the proper authorization.

However, further analyses suggest that these are not laboratory artifacts. If chimeras arise in library construction, they should increase as the prevalence of bacterial DNA/RNA increases. Therefore, we evaluated the possibility of a correlation between the number of read pairs arising from the *Pseudomonas*-like DNA and putative LGT read pairs for these six STAD samples. The Spearman-rank correlation between these values was not significantly different from zero (P = 0.19), indicating no correlation between the abundance of reads from the bacteria genome and reads supporting integration of *Pseudomonas*-like DNA in the human genome for these six samples. Overall, no relationship was observed between bacterial integrations and (a) the microbiome composition, (b) human transcript abundance, or (c) mitochondrial transcript abundance.

Yet, to further examine laboratory artifact chimerism in these samples, the distribution of the insert sizes for paired reads was compared between representative LAML samples, STAD samples, and *Neisseria meningitidis* whole genome sequencing project samples (Figure S9). The mappings with *N. meningitidis* were used to establish that ∼0.22–0.29% of reads were outside this distribution when the reads were mapped to the assembled genome for that exact strain. For comparison, 0.94–1.12% of reads were outside this distribution when reads mapped to a divergent genome from the same species (Table S5). The same percentage values for paired reads only mapping to bacteria in the STAD samples ranged from 0.06–0.60% while those for LAML ranged from 0.65–0.87% (Table S5). Given that the database we searched against was limited to only those with complete genomes, it is highly unlikely that the bacterial DNA sequenced through the TCGA is from the same strain that has a genome deposited in RefSeq. Therefore, we anticipate values should lie between 0.22–1.12% as is seen in the *N. meningitidis* controls. We observed that bacterial read pairs in the TCGA data fell outside the distribution less frequently (0.06%–0.60%). While this percentage is used as a proxy for laboratory artifactual chimerism, bacterial genomes are known to be fluid with genome rearrangements happening in single growths that would result in the same outcome. When these results are taken together, there is no indication that there is a higher level of chimerism in the bacterial DNA of TCGA samples than is normally observed. Furthermore, this level of chimerism would not explain 4× or 150× coverage across the bacterial integrations that are discussed here considering that PCR duplicates were removed. Of note, high coverage chimerism in bacterial samples would lead to an inability to properly assemble the corresponding genomes, which is not observed in microbial genome sequencing projects.

We note, however, that laboratory artifact chimeras could be detected in TCGA samples with whole genome amplification. As such these samples were eliminated from further analysis beyond what is presented in Table 1. In ovarian cancer, numerous read pairs that would normally support integrations were detected in both tumor and normal samples (Table 1). Upon further examination, all of the putative integrations involved *E. coli* DNA and are likely chimeras formed during the whole genome amplification used for these samples and that formed between human genomic DNA and small pieces of *E. coli* DNA introduced with recombinant enzymes.

Further support that the putative integrations in LAML and STAD samples are not laboratory artifacts comes from the fact that reads supporting integrations were detected 672× and 13.2× more frequently, respectively, in these cancer samples than in representative non-cancer samples (Table 1). This is expected if such mutations were part of the clonally expanding tumor. Across all samples, there are 1,033 reads supporting integrations in the normal samples with 13,392,142,331 read pairs sequenced, yielding an estimated integration frequency of 7.7×10^−8^. In contrast, 690,528 read pairs support integrations in tumor samples out of 42,533,195,146 paired reads sequenced, yielding an integration frequency of 1.6×10^−5^, or 210× higher. Even when compared to the highest integration rate assessed in normal samples, which was 6.02×10^−6^ in the Trace Archive data, the aggregate rate across all cancer samples is still >2.5-fold higher.

While the integration rate in cancers is 210× higher than that in normal samples across the TCGA, this comparison is not directly between matched tumor and normal pairs since normal samples were only present for OV, GBM, and BRCA. However, many different types of normal samples can be used in cancer studies and therefore other comparisons besides matched pairs are quite valid. In fact, no one type of normal sample may be perfect for all experiments. For example, a small piece of adjacent breast tissue determined to be non-cancerous by a pathologist would be considered the normal specimen for breast cancer [58]. These samples are often taken from the margins of tumors when they are resected during surgery. In that case, it's possible they could have cancer characteristics not evident by histology [59], [60]. In OV, blood-derived samples were collected as normal samples from some patients, while others had normal tissue collected. In GBM, only blood-derived samples were collected as normal samples. In other cancer studies, skin tissue from patients prior to treatment may be used [61]. Some blood cancers lack a normal sample because the cancer originates in the bone marrow. Therefore, all of the patient's blood contains cancerous cells [62]. In this instance either blood from healthy individuals [63] or blood taken from the patient once in complete remission [64] may be used as a normal sample.

Unfortunately, the STAD and LAML samples of greatest interest here for driving the dramatically increased integration rate in the tumor samples also lack normal matched samples in this data release. Given the lack of normal matched samples, and that blood samples from healthy individuals are frequently used as normal samples for studying types of leukemia [63], it is informative to compare LAML to normal samples from OV, GBM, and BRCA or 1000 Genomes data. Of note, the normal samples for OV, GBM, and BRCA have integration rates of 1.1×10^−7^, 2.3×10^−8^, and 1.2×10^−7^ (Table 1) respectively, while the integration rate of samples from the 1000 Genomes project was 2.3×10^−6^. Of these, the BRCA mutation rate is most relevant to STAD and LAML as all three are RNA-based sequencing. Comparing these, LAML samples have an integration rate 672× higher than the integration rate for the BRCA normal samples (Table 1). Even if the LAML cancer samples are compared to the normal samples with the highest integration rate, those in the Trace Archive, the integration rate for LAML is still almost 14× higher. While the overall integration frequency of cancer samples is 1.6×10^−5^, or 210× higher than the normal integration rate of 7.7×10^−8^ (Table 1), the LAML integration rate is the main driver of the increased frequency. Most tumor types do not have an increased integration rate relative to normal samples (Table 1).

Another main contributor to the significant increase of integrations in cancer samples is STAD, which has an integration rate of 1.6×10^−6^ and is 13.2× higher than the integration rate for the BRCA normal samples (Table 1). Considering STAD is in close proximity to the microbiome, normal stomach tissue would better reflect this exposure to the microbiome, including an increased likelihood of bacterial integration. That means it would be particularly informative if available. Unfortunately, this release of the TCGA lacks STAD normal samples or any other normal samples with constant exposure to the microbiome. This prevents us from determining the rate of integration in non-cancer cells with an abundant microbiome. Further work is needed to resolve differences in the integration rate between normal samples that have constant contact with the microbiome and those that do not.

### Bacterial integration of *Acinetobacter*-like DNA in mitochondrial genomes

The majority of the bacterial integrations detected were between an *Acinetobacter*-like organism and the mitochondrial genome. *Acinetobacter* spp. are known to invade epithelial cells and induce caspase-dependent and caspase-independent apoptosis [65]. Uptake of apoptotic bodies and caspase-dependant DNA fragmentation is known to facilitate LGT between mammalian cells [66], including LGT of oncogenes [67].

While we present this as bacterial integration into the mitochondrial genome, it is possible that mitochondrial DNA is integrating into an *Acinetobacter*-like genome. However, despite the numerous complete *Acinetobacter* genomes sequenced, mitochondrial DNA has not been detected in the genome of an *Acinetobacter* isolate.

Human mitochondria have an essential role in many key cellular processes such as the generation of cellular energy, production of reactive oxygen species, and initiation of apoptosis. The accumulation of somatic mutations in the mitochondrial genome has been implicated in carcinogenesis [68]. For instance, mutations in the mitochondrial cytochrome oxidase subunit I (COI) gene contribute to the tumorigenicity of prostate cancer through an increased production of reactive oxygen species [69]. The LGT from bacteria, such as *Acinetobacter*, to the mitochondria may be generating novel mutations in the mitochondrial genome and therefore influencing tumor progression.

### Bacterial integration in proto-oncogenes in stomach adenocarcinomas

The integrations we identified in STAD frequently appear to be in, or near, the untranslated region (UTR) of known proto-oncogenes. In this case, these proto-oncogenes are genes known to be up-regulated in cancers. Despite occurring in or near UTRs, this does not reflect similarity in these sequences. The mappings are specific as observed by both the BWA matches and BLAST searches against NT. While CEACAM5 and CEACAM6 are paralogs, they are sufficiently diverged to be resolved. We postulate that these putative integrations have mutated a repressor binding site and have induced over-expression leading to carcinogenesis. While this is a tempting speculation, it needs to be experimentally verified.

In chromosome 2, one STAD sample had an integration site in the second exon of thymosin β10 (TMSB10; Figure 8A) while another integration site was found in IGKV4-1. The TMSB10 gene has been shown by SAGE to be up-regulated in gastric tumors and confirmed with Northern blots [54].

On chromosome 19, integrations were identified in CEACAM5 and CEACAM6 of STAD tumors (Figure 8BC). The same integration site in CEACAM5 was detected in two separate samples while a third sample had a similar integration in CEACAM6. CEACAM proteins were initially identified as prominent tumor-associated antigens in human colon cancer [55]. Approximately 50% of human tumors show over-expression of CEA family proteins [56]. CEACAM5 and CEACAM6 mediate cell adhesion by binding to themselves and other CEACAM family members [55]. Over-expression disturbs ordered tissue formation in 3D tissue culture and leads to increased tumor formation in mice [55].

On chromosome 5, integration sites were identified in STAD tumors in different portions of CD74 with three samples having an integration in the 5′-end of the gene and one of those samples having a second integration in the 3′-UTR of CD74 (Figure 8D). CD74 initiates antigen presentation as well as signaling cascades that result in cell survival. Therefore it is not surprising that while its regulation is tightly controlled in normal tissues, it has increased expression in many cancers including gastrointestinal carcinomas and precancerous pancreatic lesions [57].

Importantly, and significantly, we only identified integrations meeting our criteria in these 4 tumor-associated genes and one other immune-related gene. We did not first look at all known oncogenes and try to find bacterial integration with these criteria, nor did we look at oncogenes and try to explain why they are up-regulated. These four oncogenes merely emerged as those having such integrations.

While there is an association between bacterial DNA integration and up-regulation of these genes, it is important to note that LGT is not associated with the most abundant bacterial transcripts. Such a result would be expected if the read pairs were merely laboratory-based artifactual chimeras generated during library construction. While these human transcripts are up-regulated in the tumors when compared to other tumors, in at least two cases they are not the most abundant transcripts. In fact, in the 143 STAD samples, if we examine the most abundant transcript, it is most frequently annexin A2 (Table 3), which was not identified as having a bacterial integration. Using our search criteria, we find no evidence of human-bacteria chimeras in any of the most abundant transcripts (Table 3) that would suggest such sequences arise from laboratory artifacts. If we, instead, focus only on the abundance of the four up-regulated genes above and on the ten samples where we identified bacterial integration in these genes, we see no clear pattern that would correlate LGT with transcript abundance. In CEACAM5, which has the most bacterial integrations, and CEACAM6, they are >75% less abundant than the most abundant transcript in that sample (Table S6). In addition, there are between 35 and 95 transcripts that are more abundant depending on the sample examined (Table 4).

**Table 3:** Most abundant transcripts across all 147 STAD samples.

Count	Accession	Gene
48	NR_003573	Annexin A2 psuedogene 2, (ANXA2P2), non-coding RNA
25	NM_013230	CD24 molecule (CD24)
16	NM_000146	Ferritin
9	NR_037688	Actin, gamma 1 (ACTG1), transcript variant 3, non-coding RNA

**Table 4:** Transcript and rank abundance for the four STAD transcripts with LGT.

Gene	Accession	Sample A	Sample B	Sample C	Sample D	Sample E	Sample F	Sample G	Sample H	Sample I	Sample J	Sample K
**CEACAM5**	**NM_004363**	9.4	9.6	7.8	9.2	<0.1%	<0.1%	1.1	2.4	2.6	<0.1%	<0.1%
		(92) *	(89)	(64)	(61)	(20,972)	(21,922)	(999)	(309)	(329)	(16,983)	(17,174)
**CEACAM6**	**NM_002483**	4.1	4.4	5.2	6.7	0.6	0.5	0.4	12.6	13.6	<0.1%	<0.1%
		(289)	(281)	(129)	(96)	(1,422)	(1,586)	(2,610)	(36)	(38)	(20,518)	(23,082)
**Thymosin B10**	**NM_021103**	44.8	43.4	41.9	41.7	64	60.3	70.8	39.3	42.4	57.3	48
		(15)	(14)	(9)	(10)	(3)	(3)	(8)	(9)	(9)	(2)	(5)
**CD74**	**NM_001025159**	16.5	17.6	4.7	5.5	41.1	32.5	38.2	16.4	18.7	53.2	49.7
		(46)	(45)	(140)	(132)	(12)	(15)	(14)	(25)	(23)	(4)	(3)

* Transcript abundance was measured by taking the RPKM for that gene in that sample and dividing it by the RPKM of the most abundant gene in that sample; the rank abundance is shown in parentheses. Values that are underlined are from transcripts that have evidence of bacterial integrations in that sample.

Furthermore, multiple samples have bacterial integrations in CEACAM6, but not the more abundant thymosin β10. In fact, thymosin β10 is more abundant in all of these samples, yet we detect integrations in thymosin β10 only in one of the samples (Table 4). If these genes were somehow primed to participate more in forming chimeras (e.g. through sequence similarity between the bacteria and human genes or by having an altered 5′-cap), one would expect that putative integrations would be frequently associated with thymosin β10, and this is not observed.

Identification of both sides of an integration is powerful evidence that these are not merely laboratory artifacts. In the Trace Archive data, such clones would contain a read with non-overlapping similarity to human and then bacterial sequences and the other read would have similarity to a human read. These clones would contain a bacterial sequence flanked on both ends with human sequence in a single clone. Additionally, they should not be detected as frequently as clones with one bacterial read and one human read. Consistent with this we find 2 clones, out of 319 clones, with these characteristics. In the 1000 Genomes projects where the coverage of all reads sequenced across the human genome was often less than 1× coverage, we were unable to accurately find both sides of the integration. In LAML, the integrations were primarily found randomly distributed around the human mitochondrial genome. While many putative pairs of paired reads can be identified that may constitute both sides of the integration, the large number of putative transfers and the presence of multiple mitochondrial genomes precludes this assignment of pairs flanking integrations with any confidence. It is unlikely that both sides of the integrations would be found in the STAD RNA sequencing project because the integrations were found to be in the 5′-UTR and the remaining piece would be quite small relative to the library insert size and be declining in abundance due to the nature of sequence data at the ends of transcripts.

### Integration of bacterial rRNA

If we examine the bacterial portion of the transcript, it is frequently rRNA. There are at least two possible explanations for this observation, including that (a) rRNA is easier to detect in samples because regions in the rRNA gene are conserved across all bacteria, and (b) bacterial rRNA actually integrates more frequently. The former can be excluded as a possibility since in LAML 68% of the microbiome reads were from rRNA yet 99% of the LGT reads were from rRNA. This suggests that bacterial rRNA actually integrates more frequently than other RNA.

Integration of bacterial rRNA is consistent with our understanding of the nucleotides recognized by the human innate immune system. Unmethylated CpG DNA [70] and mRNA [71] from bacteria are both recognized by the innate immune system, but at least some rRNA is not [71], [72]. Some rRNA is detected by the immune system [72], possibly explaining why not all bacterial rRNA mutagenizes the human genome. As such, immune response may prevent integration through DNA or mRNA intermediates, but be permissive to the integration of some rRNA.

There is also a precedent for integration of rRNA into animal genomes that suggests the mechanism of bacterial integration. SINE elements are derived from tRNA [73], 7SL rRNA [74], and 5S rRNA [75] and are integrated via retrotransposition using endogenous retrotransposon machinery. It seems plausible that bacterial rRNA and tRNA may also be integrated using the same machinery. However, the mechanism by which DNA/RNA enters the human cell is not as readily apparent.

### Barriers to describing LGT

There continue to be several barriers to the description of LGT using only genome sequencing data. The prevailing paradigm is to assume laboratory artifacts when other experimental evidence is lacking. Maintaining this status quo ensures that LGT in eukaryotes will continue to be overlooked and under-estimated. There is a notion that this is necessary in order to avoid LGT from being described inappropriately. This notion, as well as high profile erroneous reports of LGT in humans and other animals (e.g. [7], [8], [76]–[78]), has had a chilling effect on the field.

Ironically though, experimental validation of LGT is usually in the form of PCR amplification (e.g. [15], [17], [79], which is also the potential source of such artifacts in current sequencing protocols. While PCR amplification is an independent validation of capillary sequencing, it is not an independent validation of next generation sequencing data. One way chimeras are introduced in Illumina sequencing data is during sequencing library preparation through cDNA synthesis for RNA samples and PCR amplification for both RNA and DNA samples [47]. Yet validation of LGT would occur through cDNA synthesis and/or PCR amplification. Except for Northern blots, most experimental RNA work proceeds through a cDNA synthesis step making that step difficult to avoid. Regardless, experiments that include cDNA synthesis or PCR amplification should not be considered independent validations of next generation sequencing data.

Arguably, such experimental validation is not necessary with newer and more sophisticated methods like those used here. One of the most prominent reasons for needing experimental validation of genome sequencing has been due to errors made by assembly algorithms. Such errors result in the erroneous joining of two pieces of a genome into one piece with little sequence support (e.g. a single read spanning a small segment with limited similarity). These errors could be assessed by examining the assemblies themselves for coverage and read quality that would suggest missassembly in a region. However, few researchers had access to that assembly data and it was often limited to the generators of the assemblies. While such files could be deposited in NCBI's Assembly Archive [80], this was infrequently done. For example, as of April 01, 2013, there were only 193 bacteria and 31 eukaryotes with assemblies in the Assembly Archive while there were 18,756 bacteria and 3,017 eukaryote with genome projects registered at NCBI. Researchers without access to such assembly data have needed to experimentally validate the sequence/structure of specific contigs in genome sequences. In our experience, if the underlying assembly was examined for well supported junctions, one was 100% successful in subsequent experimental validation of such bacterial integrations into animal genomes using just the assembly data. Therefore, we designed our current analysis in a manner that does not rely on assembly. It relies instead on sequence read mapping with an emphasis on coverage, indicating higher support across junctions of bacterial and human DNA.

### Conclusions

Populations of human cells have a constant, intimate relationship with the human microbiome. With that comes a potential for LGT that could be analogous to disease-causing DNA insertions by transposons, retroviruses, or mitochondria. Although chronic inflammation is increasingly implicated as a mechanism for cancer development following bacterial infection, proto-oncogene disruption by bacterial DNA could provide yet another mechanism. A well-established model for bacterial disease induced through somatic cell LGT was described many years ago, namely *A. tumefaciens* induced crown gall disease in plants. As nature often repeats itself, the results presented here indicate a similar situation may be applicable to humans and warrant targeted research projects aimed at identifying LGT from the microbiome to human somatic cells.

Taken together, putative integrations of bacterial DNA in human tissues, including tumors, can be detected with next-generation sequencing. Such integrations were detected 210× more frequently in tumor samples than normal samples. Putative integration sites in known cancer-related genes were identified with >4× coverage on the human genome. With the currently available datasets, such integrations are most frequently detected between bacterial rRNA and cancer samples from acute myeloid leukemia and stomach adenocarcinoma. While it is tempting to speculate that integration of bacterial DNA may cause cancer, particularly given the detection of integrations in oncogenes that are over-expressed in these samples and the detection of the same integrations in multiple individuals, further carefully controlled experiments are needed, but now justified.

## Materials and Methods

### Trace Archive analysis

The 113,046,604 human shotgun sequencing traces in the NCBI Trace Archive as of March 11, 2009, were compared to all the bacterial genomes available on the NCBI genomes ftp site on November 11, 2010. Initial matches between these two datasets were identified with NUCMER using the MAXMATCH parameter [37]. A data subset was then created of the human traces with positive matches and all other reads from that clone using the XML available from NCBI parsed with custom scripts. This data subset was searched against NT using BLASTN [81]. The output of these BLAST searches was parsed to identify bacterial DNA linking to human DNA either directly or within a clone. The corresponding chromatograms hosted at NCBI and the wwwblast results against NT for 2,871 sequence pairs were inspected manually to remove poor quality sequences, vector contaminants, and low complexity sequence matches resulting in a curated set of putative integrations (Table S1, S2). Importantly, the traces found to contain an integration boundary within the trace may also contain an integration boundary measured within the clone. In this way, the two counts are not exclusive of one another.

### Analysis of Illumina data from the TCGA

Illumina sequences were downloaded from the 1000 Genomes Project that were in the NCBI Short Read Archive as of November 2010 and from the TCGA in the NCBI dbGap between September 18, 2011, and September 20, 2011. All reads were mapped to both the human genome (hg19) and all the bacterial genomes available on the NCBI genomes ftp site on November 11, 2010 using the short read mapper BWA [40] with the default parameters. Using custom scripts, pairs of reads were identified as spanning integrations when only one read mapped to the human genome and its mate mapped to a bacterial genome.

Unless otherwise noted, paired reads spanning junctions that were identified in the initial BWA screen were screened for uniqueness, low complexity, and taxonomy. Low complexity sequence and duplicate reads were removed using PRINSEQ [82]. For low complexity filtering, the DUST method with an entropy cutoff of 7 was applied to each read in a pair separately. A pair is considered low-complexity if either read is considered low complexity. Duplicate reads were flagged by concatenating the two reads together in a pair and running the PRINSEQ derep function to find exact duplicates and the reverse complements of exact duplicates (flag 14). After low complexity and duplicate screening, both bacterial and human reads were searched against NCBI's NT database using BLASTN with an e-value cutoff of 10^−5^. Reads identified as bacterial in the initial BWA screen were required to match bacteria in NT and not have a best match to human. The bacterial half of all putative LGT's was remapped against all complete bacterial genomes in RefSeq individually. These mappings were used to assign an OTU based on the LCA. The microbial composition was examined using Krona plots [83] as well as heat maps generated in the R software package.

BWA computes were executed using the CloVR virtual machine [84]. The CloVR virtual machines were deployed in parallel on the Data Intensive Academic Grid (DIAG) cloud infrastructure. Data staging, output retrieval and cluster management was accomplished using CloVR's Vappio software package.

### Assignment of lowest common ancestor

Reads identified as putative bacterial reads in either the microbiome or lateral gene transfer were mapped using BWA with default parameters against all complete bacterial genomes in RefSeq. The LCA is calculated based on the congruent taxonomy for all genomes with mappings. The use of RefSeq limits the taxonomic assignments available to only those with complete genomes. However, the use of genomic sequences, as opposed to all deposited sequences in NT, ensures that the taxonomic assessment of the database sequence is correct. For reads assigned to the microbiome, once the LCA is calculated, the most specific taxonomic assignment is used as the bacterial OTU (Figure S10).

### Generation of circular figures

Circular figures were generated with Circos [85] using putative LGT reads filtered using the method described above. Down sampling of the data to 5% for Figure S4A, 0.5% for Figure 6A and 2% for Figure 6BC was needed to successfully draw the purple linkages. For Figure 3 the data was not blast-verified in the same manner as the bacterial integration data as there are significant amounts of HPV integrant sequence data in the database with human listed as the source in the taxonomy. While this is correct, it stymied the blast validation. Therefore, reads were blast validated to confirm that they were HPV – human, but they could also be human – human in this screen with one of the human reads arising from HPV since many HPV reads exist in reference databases with the taxonomy assignment of *Homo sapiens*.

### Coverage measurements

Each read pair was assigned an average coverage value measured along the human mapping that was used to hone in on integrations with increased coverage. This value is obtained by running samtools mpileup [86] on the human read for each read pair indicating an integration. Coverage was calculated separately for each sequencing run. If a human read was assigned a value of >4× coverage, it had at least five unique reads aligning to that region on the human genome. Reads on the integration site cannot be mapped with BWA, but would be adjacent to reads supporting that integration.

### Expression analysis

The RNA-Seq reads were mapped against hg19 with BWA and the reads per kilobase of gene per million human mapped reads (RPKM) was calculated as using the predicted transcriptional start and stop sites available from the UCSC annotation. The ratio was calculated by dividing the RPKM for a given gene in a given run by the average RPKM for that gene across all runs. The log_2_ratio was used for the expression analysis presented in Figure 9.

### Generation of phylogenetic trees

Nine randomly selected reads supporting bacterial integrations were searched against the NT database using BLASTN [81] with an expected threshold of 10^−11^ and with uncultured bacterial sequences removed using the BLAST interface. Each read and its first hit for each high scoring pair was aligned in a multiple sequence alignment using ClustalW [87] with default settings. This multiple sequence alignment was then used to draw a phylogenetic tree using PhyML [88] with default settings and 1000 bootstraps. The most likely tree and bootstrap support values from PhyML were visualized using FigTree (http://tree.bio.ed.ac.uk/software/figtree/).

### Statistics

Statistical modeling and correlation analysis was performed using the R package (v 2.7.2).

## Supporting Information

Figure S1 **Detailed schematic of method employed to identify putative LGT reads.** Following the identification of putative LGT reads and microbiome reads, a series of steps were undertaken to remove low complexity sequences, remove duplicates, remap the reads, and generate data for the interfaces provided. Such data includes the assignment of an LCA, measuring coverage, and establishing overlaps with genes as well as generating krona plots and heat maps. Where possible, existing tools were used like BWA, BLAST, MPILEUP, and PRINSEQ.
(PDF)

Figure S2 **Specificity of taxonomic assignment varies according to the conserved and variable regions of the 16S rRNA.** When reads supporting bacterial integration in LAML (A) or STAD (B) were mapped to a representative *Acinetobacter* or *Pseudomonas* rRNA, respectively, the specificity of the OTU assignment tracks with the known variable regions in the 16S rRNA. This is illustrated with a bar chart where each nucleotide position is represented by a bar colored by the proportion of OTUs supported by reads aligning at that position. For example, one can observe that in the conserved regions between V2 and V3 or between V5 and V6 that OTUs are most frequently only as specific as “Bacteria”. In contrast, in the V1–V2 region more specific genus-, species-, and strain-level assignments can be made.
(PDF)

Figure S3 **Phylogenetic evaluation of BWA and BLAST LCA assignments.** Ten randomly selected reads with OTU assignments across 4 levels of the taxonomy (i.e. strain, species, genus, family) were selected for a phylogenetic analysis (Panels A–J). This analysis demonstrates parsimony between the BLAST-based OTU, the BWA-based OTU, and the phylogeny. It also higlights issues with using NT. In the release of NT used for the phylogeny, but not the initial screen, several sequences appear from eukaryotic genome sequencing projects. For example, sequences were identified with BLAST that were attributed to fish (C), moths (G), and oomycetes (H). For at least the moth and fish, it seems reasonable that the contigs generated from random sequencing and assembly may include bacterial contigs from members of the microbiome. In addition, sequences from clones of NotI digested human cell line DNA [52] appear in this analysis (J). This occurs because sequences attributed to clones were not excluded from this analysis as they were in the prior BLAST-based LCA analysis. In the manuscript describing the NotI clones, the authors suggest they are likely of *Pseudomonas* origin and represent integrations in the human genome [52] analogous to ones described here.
(PDF)

Figure S4 **Distribution of putative LGTs from the 1000 Genomes Project.** The read pairs supporting LGT (purple) into the human genome (blue) from the bacterial genome (red) with similarity to *Bradyrhizobium* sp. BTAi1 (NC_008475.1, Panel A) and *Stenotrophomonas maltophilia* K279a (NC_010943.1, Panel B) are randomly distributed across both bacterial genomes.
(EPS)

Figure S5 **Distribution of bacterial OTUs from the microbiome and bacterial DNA integrations in the 1000 Genomes Project.** The proportion of reads from each bacterial OTU is illustrated from the microbiome (Panel A) and LGT (Panel B) across the 1000 Genomes Project. The log-transformed proportion of bacterial OTU per sample for the microbiome (Panel C) and LGT (Panel D) are clustered based on the microbiome profiles in Panel C and illustrated using heat maps.
(TIF)

Figure S6 **Histogram of the coverage across the human genome resulting from the aggregate of all reads supporting bacterial integration in the TCGA.** The frequency of positions in the human genome is illustrated relative to a given coverage supporting bacterial integrations.
(EPS)

Figure S7 **Distribution of bacterial OTUs from the microbiome and bacterial DNA integrations in acute myeloid leukemia.** The proportion of reads from each bacterial OTU is illustrated from the microbiome (Panel A) and LGT (Panel B) across all LAML samples. The log-transformed proportion of each bacterial OTU per sample representing the microbiome (Panel C) and LGT with >4× coverage (Panel D) are clustered based on the microbiome profiles in Panel C and illustrated using heat maps.
(EPS)

Figure S8 ***Pseudomonas*** **-like integrations into the genome of stomach adenocarcinoma samples.** Putative integrations into the human nuclear genome (blue) from a *Pseudomonas* level OTU (red) in stomach adenocarcinoma are illustrated. Only reads mapping to regions of the human genome with >4× coverage are shown.
(EPS)

Figure S9 **Histograms of the insert size of paired reads mapping to the bacterial genome.** The distribution of the insert sizes was calculated from the paired reads where both reads map to the bacterial genome for four STAD samples (Panels A–D) and four LAML samples (Panels E–H). For comparison, the distribution of insert sizes was also calculated for four *N. meningitidis* samples sequenced independently with mapping to the consensus sequence for that strain (Panels I–L) and to FAM18 (NC_008767.1) [89], a different strain of the same species (Panels M–P). For all panels the frequency of a given insert size is 1000× the y-axis value.
(EPS)

Figure S10 **Calculating bacterial LCAs and OTUs.** Reads identified as bacterial were mapped using BWA default parameters against all complete bacterial genomes in RefSeq. BWA aligns reads to the reference genomes allowing a fixed number of differences between the query read and reference genome, dependent on the read length. For example, the LAML and STAD reads are 51 bp and allowed 3 differences. After BWA has mapped the bacterial read to all bacterial genomes, an LCA is calculated based on the congruent taxonomy for all genomes with mappings. Once the LCA is calculated, the most specific taxonomic assignment is used as the bacterial OTU. This method of OTU assignment is a computationally efficient method for analyzing the human microbiome; however, the analysis is limited to the bacteria available in the database. To compensate for this limitation, a relatively conservative approach for assigning OTUs was used such that a match that contains 3-differences is weighted the same as a match with no mismatches in making the OTU assignment.
(EPS)

Table S1 **Reads from the Trace Archive supporting LGT in the somatic human genome.** The reads listed here contain non-overlapping matches to both human and bacteria sequences. Data deposited in various fields in the Trace Archive are presented along with a with a summary of the results generated.
(XLSX)

Table S2 **Read pairs from the Trace Archive supporting LGT in the somatic human genome.** The read pairs listed here have one read that matches human sequences and one read that matches bacteria sequences. Data deposited in various fields in the Trace Archive are presented along with a summary of the results generated.
(XLSX)

Table S3 **Read pairs from the 1000 Genomes project that support LGT in the somatic human genome.** The read pairs listed here have one read that matches human sequences and one read that matches bacteria sequences. Data deposited in various fields in the Sequence Read Archive are presented along with a summary of the results generated.
(XLSX)

Table S4 **Read pairs from TCGA that support LGT in the somatic human genome.** The read pairs listed here have one read that matches human sequences and one read that matches bacteria sequences. Data deposited in various fields in the Sequence Read Archive or the TCGA portal are presented along with a summary of the results generated.
(ZIP)

Table S5 **Abnormal insert sizes for Illumina libraries.** The percentage of reads with abnormal insert sizes for experimental and control samples is listed for the data presented in Figure S9.
(PDF)

Table S6 **Expression analysis for STAD samples.** The RPKM is calculated for each gene using the STAD RNAseq data.
(ZIP)
