# Supplementary figures and images for: Bacteria-Human Somatic Cell Lateral Gene Transfer Is Enriched in Cancer Samples
Source: PLoS Comput Biol. 2013 Jun 20;9(6):e1003107. doi: 10.1371/journal.pcbi.1003107 (PMC3688693; doi:10.1371/journal.pcbi.1003107)

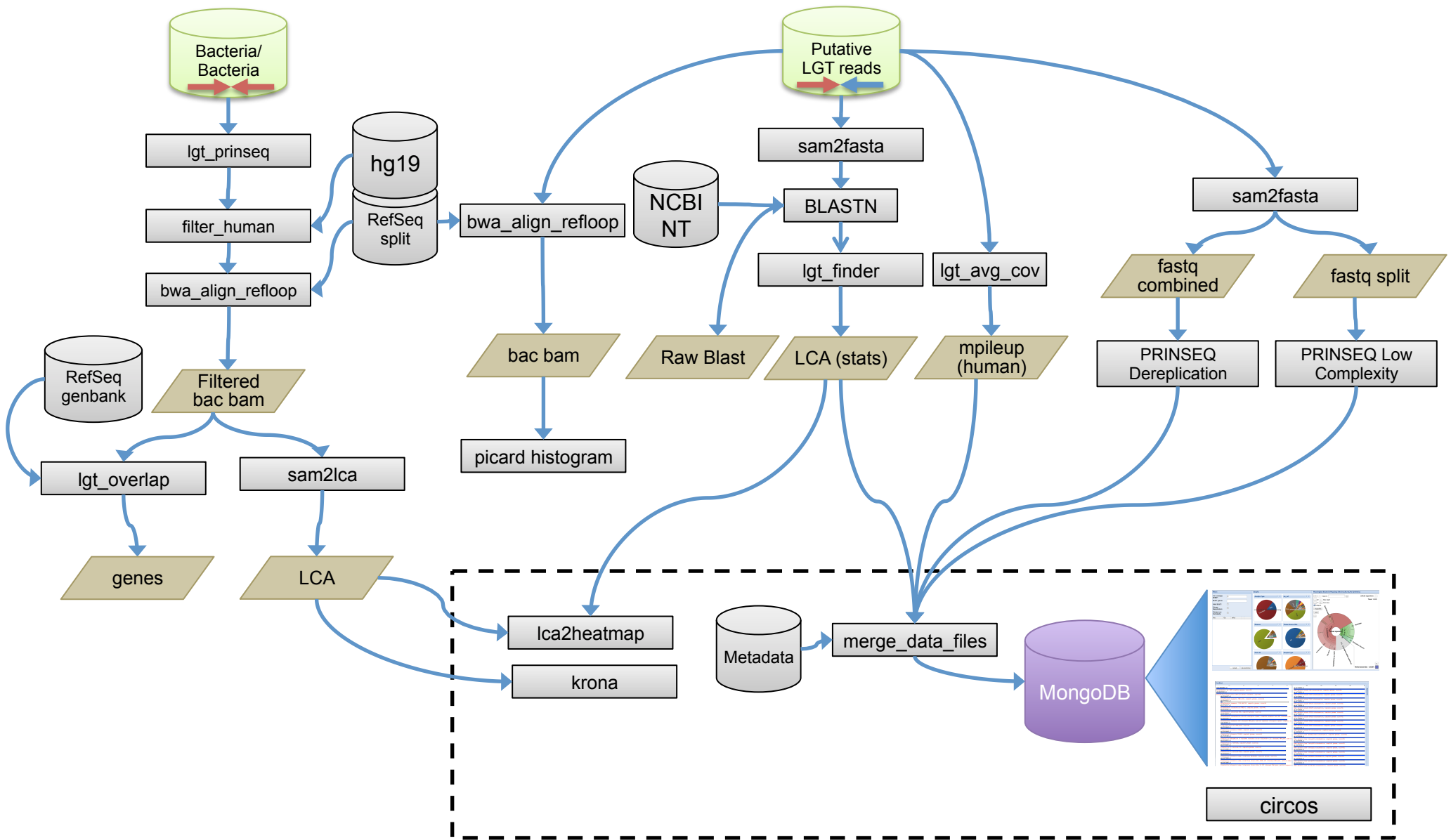

Supplement: Figure S1 — Detailed schematic of method employed to identify putative LGT reads. Following the identification of putative LGT reads and microbiome reads, a series of steps were undertaken to remove low complexity sequences, remove duplicates, remap the reads, and generate data for the interfaces provided. Such data includes the assignment of an LCA, measuring coverage, and establishing overlaps with genes as well as generating krona plots and heat maps. Where possible, existing tools were used like BWA, BLAST, MPILEUP, and PRINSEQ. (PDF) [file pcbi.1003107.s001.pdf]

# A

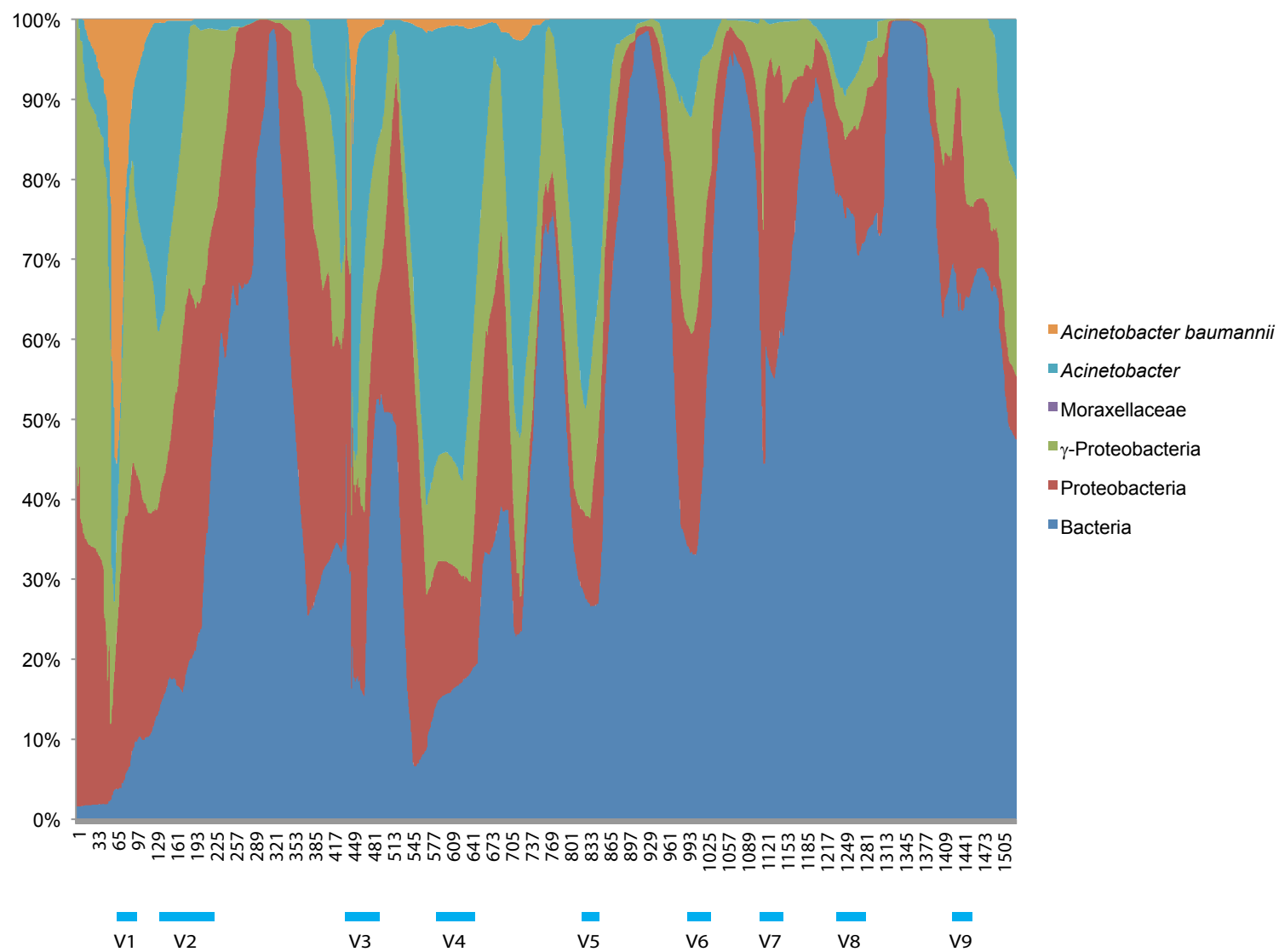

# B

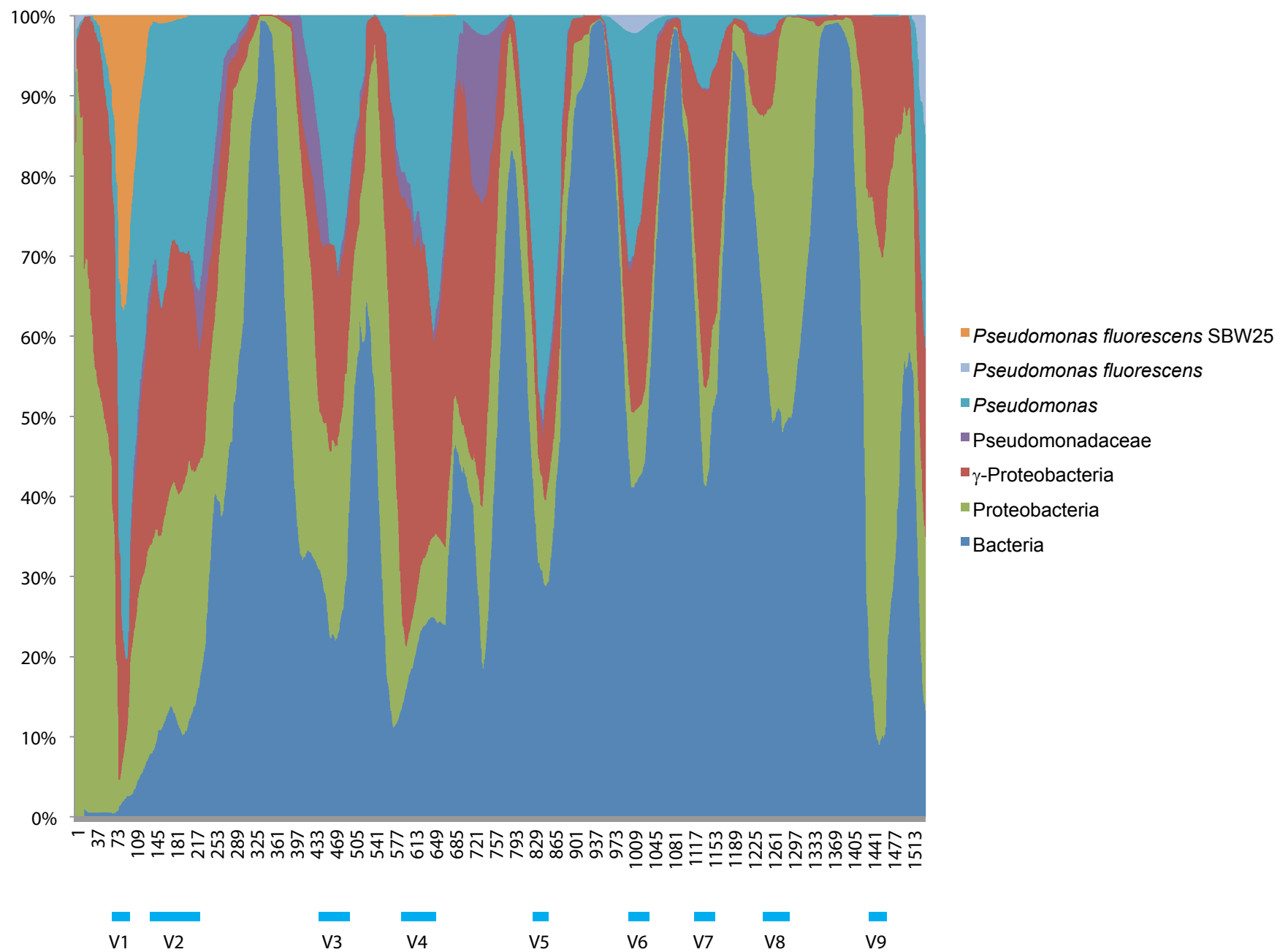

Supplement: Figure S2 — Specificity of taxonomic assignment varies according to the conserved and variable regions of the 16S rRNA. When reads supporting bacterial integration in LAML (A) or STAD (B) were mapped to a representative Acinetobacter or Pseudomonas rRNA, respectively, the specificity of the OTU assignment tracks with the known variable regions in the 16S rRNA. This is illustrated with a bar chart where each nucleotide position is represented by a bar colored by the proportion of OTUs supported by reads aligning at that position. For example, one can observe that in the conserved regions between V2 and V3 or between V5 and V6 that OTUs are most frequently only as specific as “Bacteria”. In contrast, in the V1–V2 region more specific genus-, species-, and strain-level assignments can be made. (PDF) [file pcbi.1003107.s002.pdf]

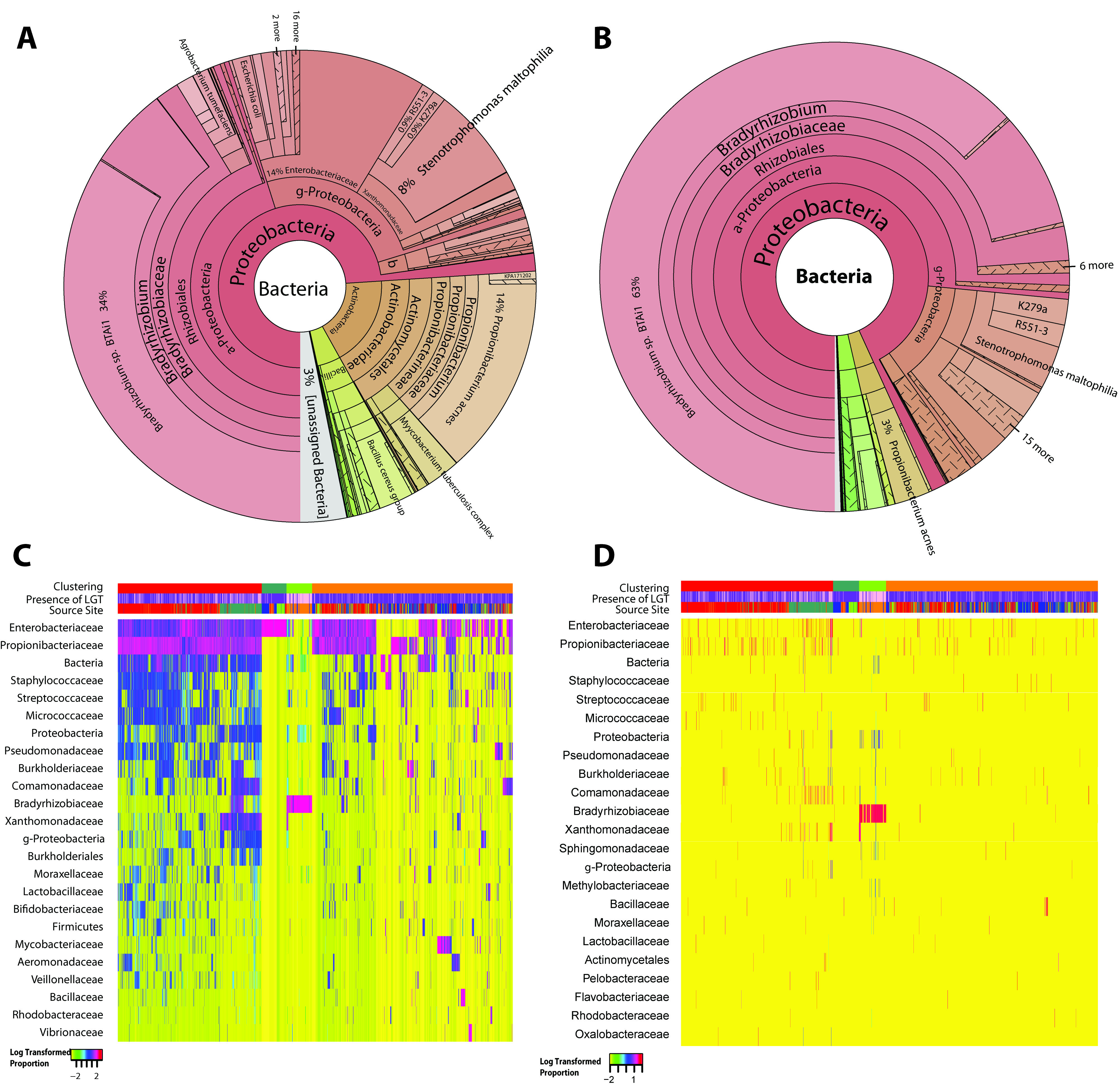

Supplement: Figure S5 — Distribution of bacterial OTUs from the microbiome and bacterial DNA integrations in the 1000 Genomes Project. The proportion of reads from each bacterial OTU is illustrated from the microbiome (Panel A) and LGT (Panel B) across the 1000 Genomes Project. The log-transformed proportion of bacterial OTU per sample for the microbiome (Panel C) and LGT (Panel D) are clustered based on the microbiome profiles in Panel C and illustrated using heat maps. (TIF) [file pcbi.1003107.s005.tif]
