# Supplementary material for: Bacteria-Human Somatic Cell Lateral Gene Transfer Is Enriched in Cancer Samples
Source: PLoS Comput Biol. 2013 Jun 20;9(6):e1003107. doi: 10.1371/journal.pcbi.1003107 (PMC3688693; doi:10.1371/journal.pcbi.1003107)
Supplement: Figure S3 — Phylogenetic evaluation of BWA and BLAST LCA assignments. Ten randomly selected reads with OTU assignments across 4 levels of the taxonomy (i.e. strain, species, genus, family) were selected for a phylogenetic analysis (Panels A–J). This analysis demonstrates parsimony between the BLAST-based OTU, the BWA-based OTU, and the phylogeny. It also higlights issues with using NT. In the release of NT used for the phylogeny, but not the initial screen, several sequences appear from eukaryotic genome sequencing projects. For example, sequences were identified with BLAST that were attributed to fish (C), moths (G), and oomycetes (H). For at least the moth and fish, it seems reasonable that the contigs generated from random sequencing and assembly may include bacterial contigs from members of the microbiome. In addition, sequences from clones of NotI digested human cell line DNA [52] appear in this analysis (J). This occurs because sequences attributed to clones were not excluded from this analysis as they were in the prior BLAST-based LCA analysis. In the manuscript describing the NotI clones, the authors suggest they are likely of Pseudomonas origin and represent integrations in the human genome [52] analogous to ones described here. (PDF) [file pcbi.1003107.s003.pdf]

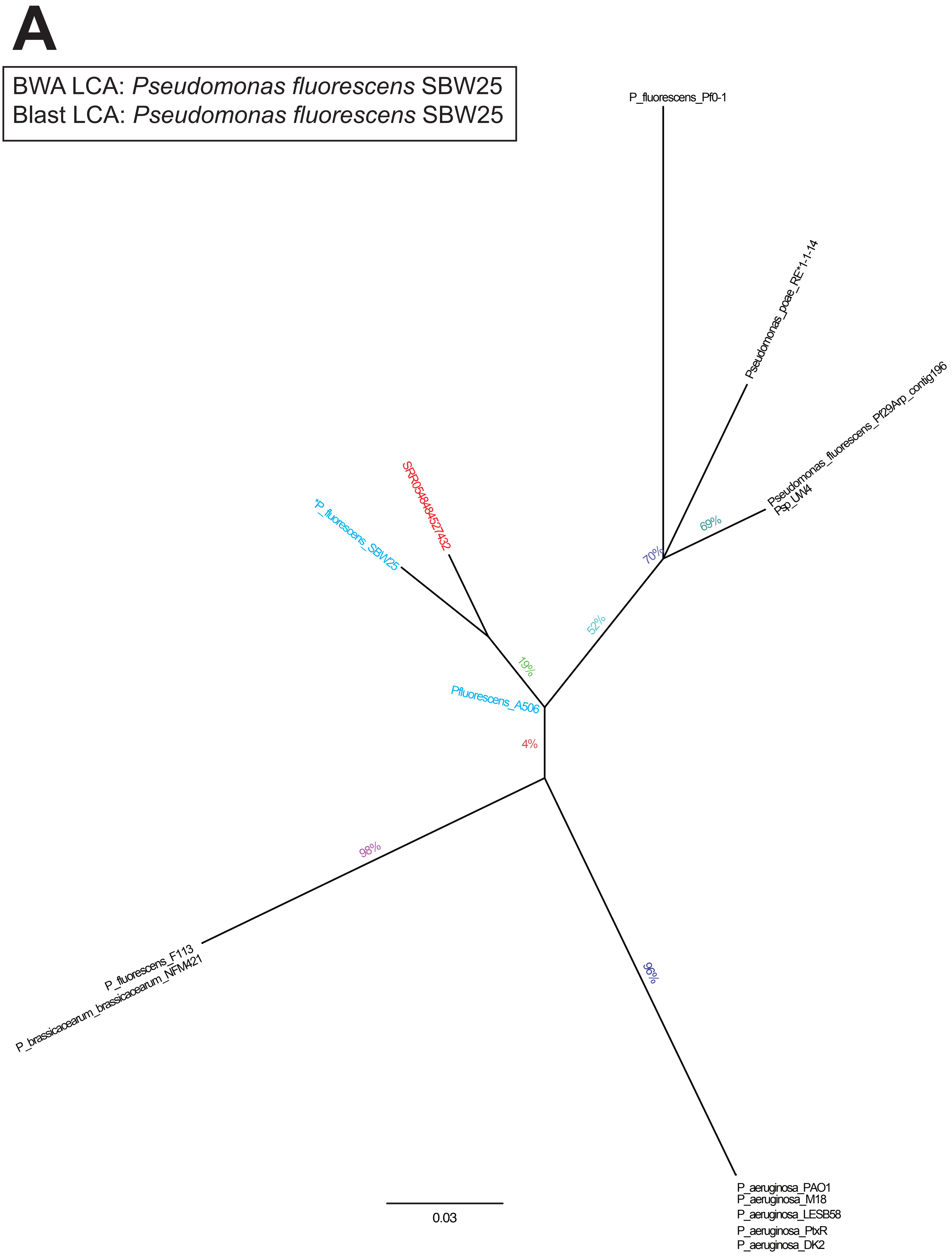

A
BWA LCA: *Pseudomonas fluorescens* SBW25
Blast LCA: *Pseudomonas fluorescens* SBW25
P_fluorescens_Pf0-1
Pseudomonas_poae_RE*1-1-14
Pseudomonas_fluorescens_Pf29Arp_contig196
Psp_UW4
69%
70%
52%
SRR054848452 7432
*P_fluorescens_SBW25
19%
Pfluorescens_A506
4%
98%
P_fluorescens_F113
P_brassicacearum_brassicacearum_NFM421
96%
P_aeruginosa_PAO1
P_aeruginosa_M18
P_aeruginosa_LESB58
P_aeruginosa_PtxR
P_aeruginosa_DK2
0.03

B

BWA LCA: *Pseudomonas fluorescens* SBW25
Blast LCA: *Pseudomonas fluorescens* SBW25

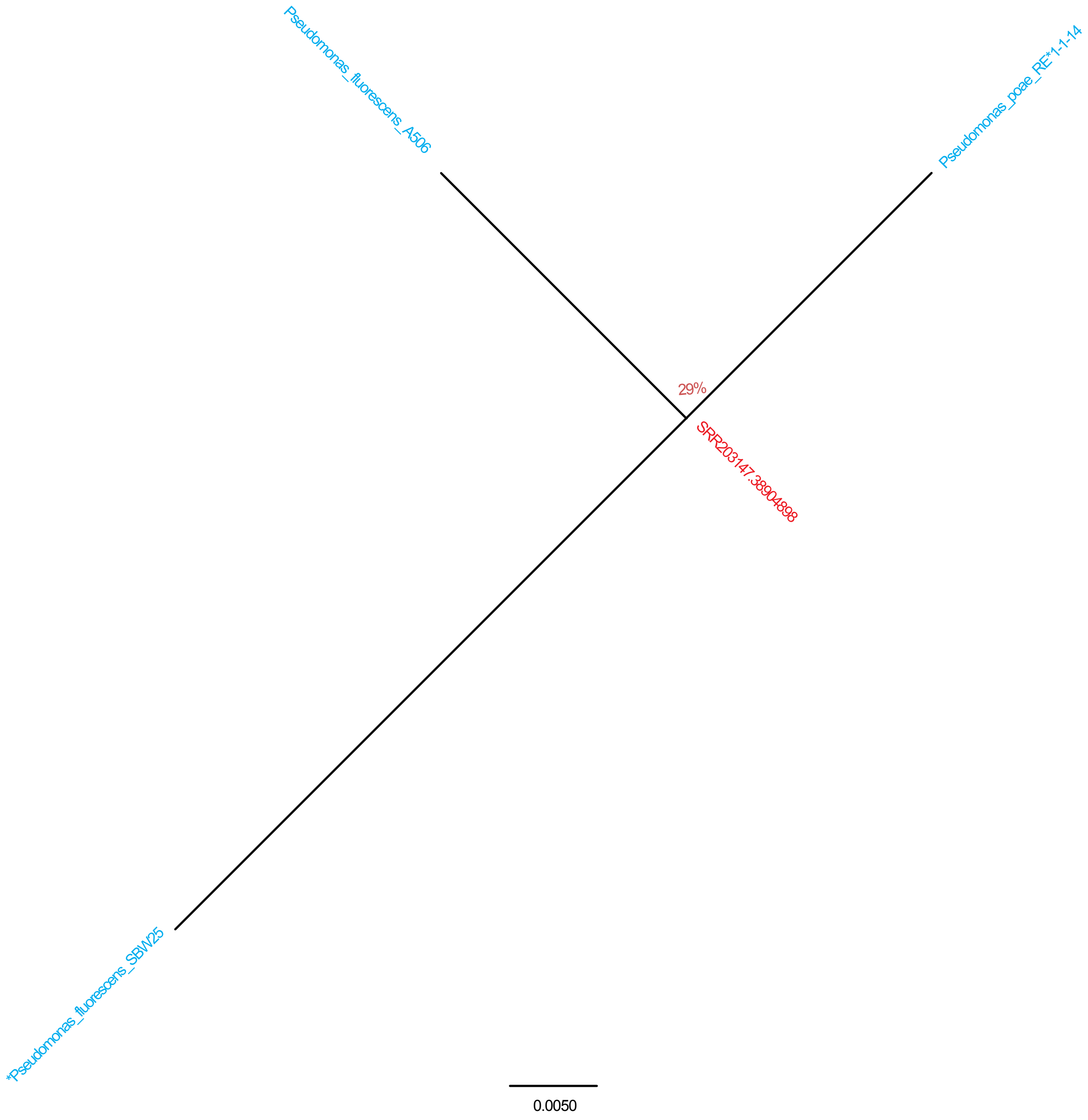

C

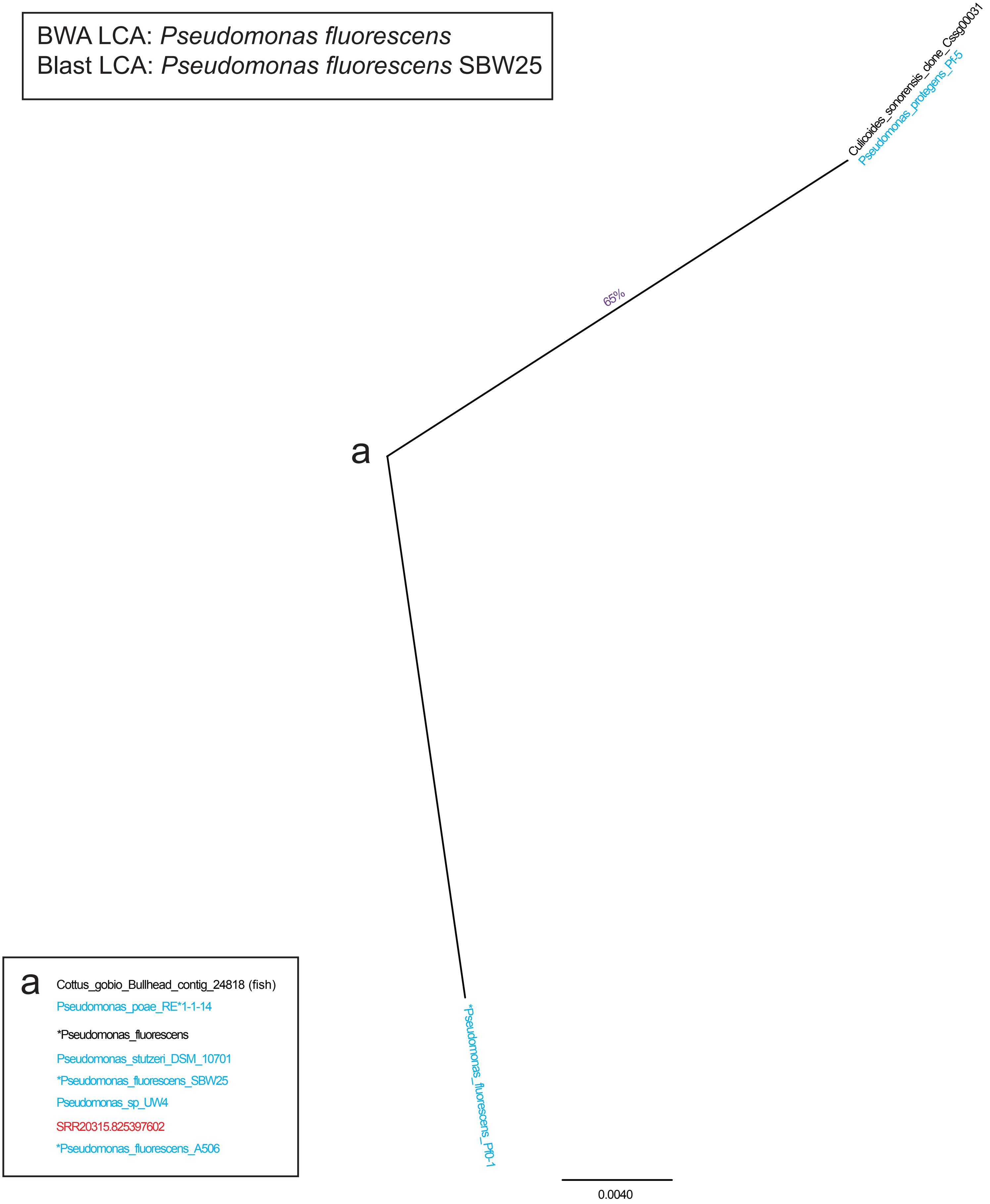
BWA LCA: Pseudomonas fluorescens
Blast LCA: Pseudomonas fluorescens SBW25
Culicoides_sonorensis_clone_Cssg00031
Pseudomonas_protegens_Pf-5
65%
a
*Pseudomonas_fluorescens_Pf0-1
0.0040
a
Cottus_gobio_Bullhead_contig_24818 (fish)
Pseudomonas_poae_RE*1-1-14
*Pseudomonas_fluorescens
Pseudomonas_stutzeri_DSM_10701
*Pseudomonas_fluorescens_SBW25
Pseudomonas_sp_UW4
SRR20315.825397602
*Pseudomonas_fluorescens_A506

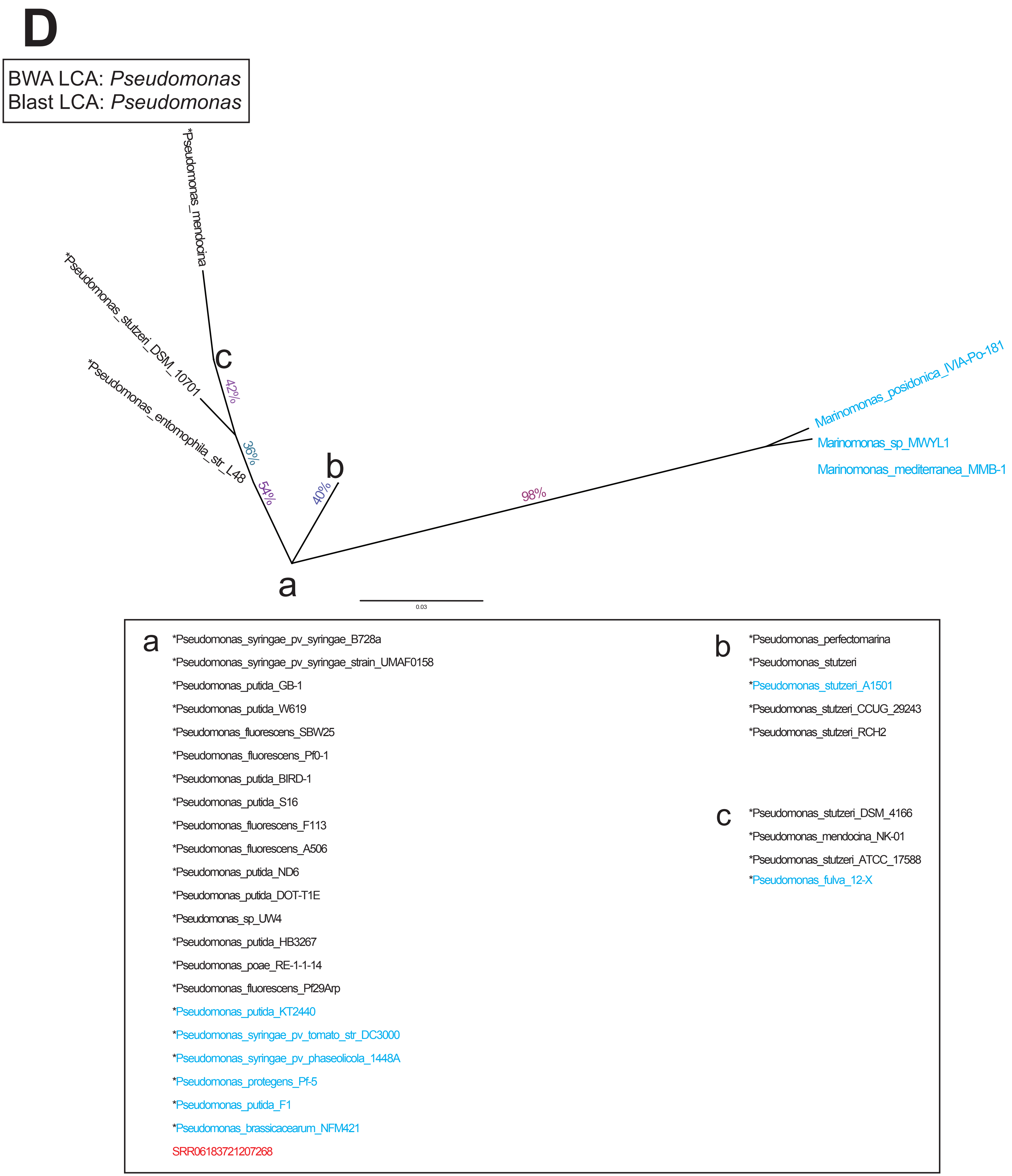
D
BWA LCA: Pseudomonas
Blast LCA: Pseudomonas
*Pseudomonas_mendocina
*Pseudomonas_stutzeri_DSM_10701
*Pseudomonas_entomophila_str_L48
c
42%
36%
54%
b
40%
98%
a
0.03
Marinomonas_posidonica_IVIA-Po-181
Marinomonas_sp_MWYL1
Marinomonas_mediterranea_MMB-1
a
*Pseudomonas_syringae_pv_syringae_B728a
*Pseudomonas_syringae_pv_syringae_strain_UMAF0158
*Pseudomonas_putida_GB-1
*Pseudomonas_putida_W619
*Pseudomonas_fluorescens_SBW25
*Pseudomonas_fluorescens_Pf0-1
*Pseudomonas_putida_BIRD-1
*Pseudomonas_putida_S16
*Pseudomonas_fluorescens_F113
*Pseudomonas_fluorescens_A506
*Pseudomonas_putida_ND6
*Pseudomonas_putida_DOT-T1E
*Pseudomonas_sp_UW4
*Pseudomonas_putida_HB3267
*Pseudomonas_poae_RE-1-1-14
*Pseudomonas_fluorescens_Pf29Arp
*Pseudomonas_putida_KT2440
*Pseudomonas_syringae_pv_tomato_str_DC3000
*Pseudomonas_syringae_pv_phaseolicola_1448A
*Pseudomonas_protegens_Pf-5
*Pseudomonas_putida_F1
*Pseudomonas_brassicacearum_NFM421
SRR06183721207268
b
*Pseudomonas_perfectomarina
*Pseudomonas_stutzeri
*Pseudomonas_stutzeri_A1501
*Pseudomonas_stutzeri_CCUG_29243
*Pseudomonas_stutzeri_RCH2
c
*Pseudomonas_stutzeri_DSM_4166
*Pseudomonas_mendocina_NK-01
*Pseudomonas_stutzeri_ATCC_17588
*Pseudomonas_fulva_12-X

E

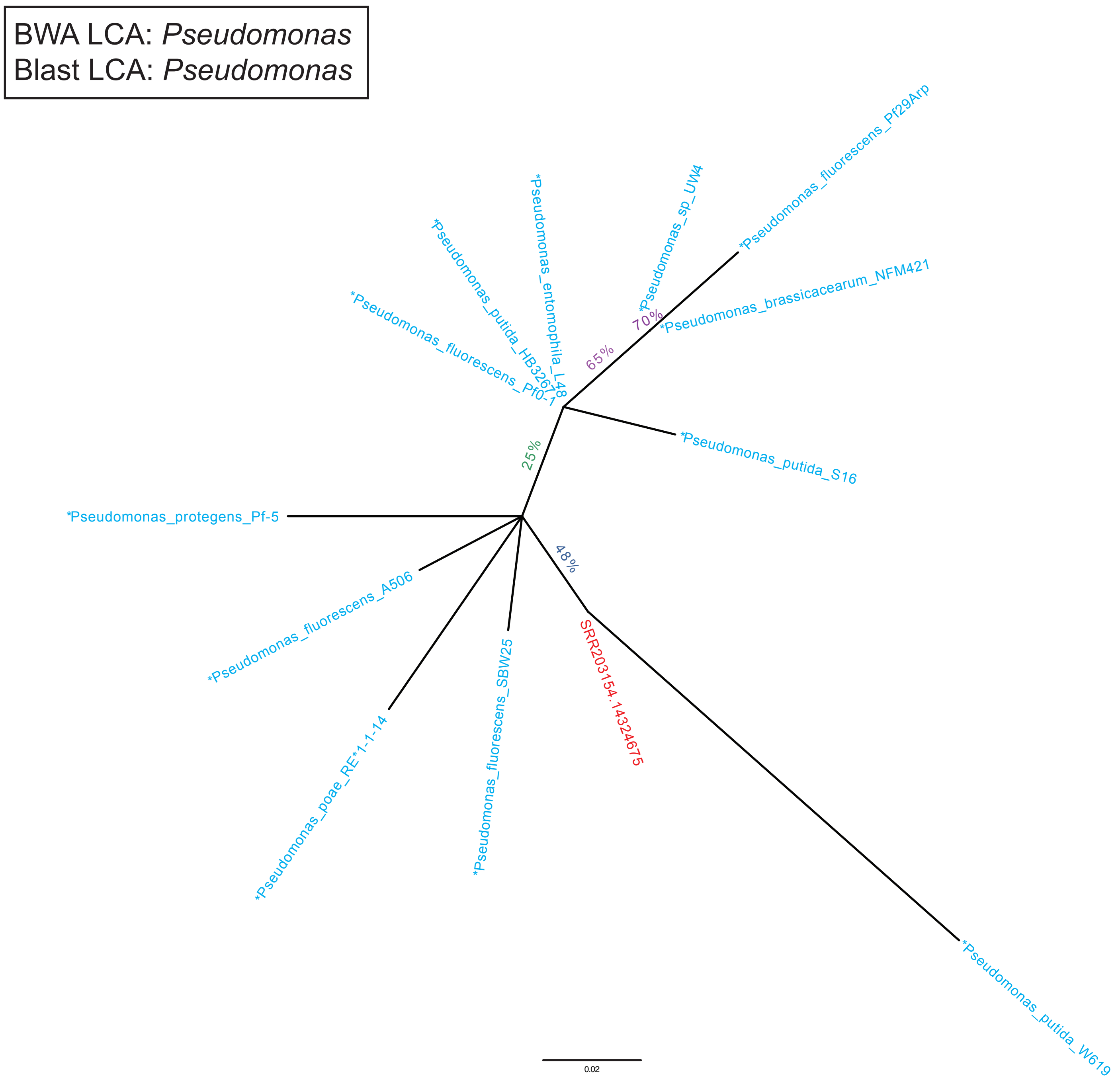
BWA LCA: *Pseudomonas*
Blast LCA: *Pseudomonas*
*Pseudomonas_fluorescens_Pf29Arp
*Pseudomonas_sp_UW4
*Pseudomonas_entomophila_L48
*Pseudomonas_putida_HB3267
*Pseudomonas_fluorescens_Pf0-1
*Pseudomonas_brassicacearum_NFM421
70%
65%
25%
*Pseudomonas_putida_S16
*Pseudomonas_protegens_Pf-5
48%
*Pseudomonas_fluorescens_A506
SRR203154.14324675
*Pseudomonas_fluorescens_SBW25
*Pseudomonas_poae_RE*1-1-14
*Pseudomonas_putida_W619
0.02

F

BWA LCA: *Pseudomonas*
Blast LCA: *Pseudomonas*

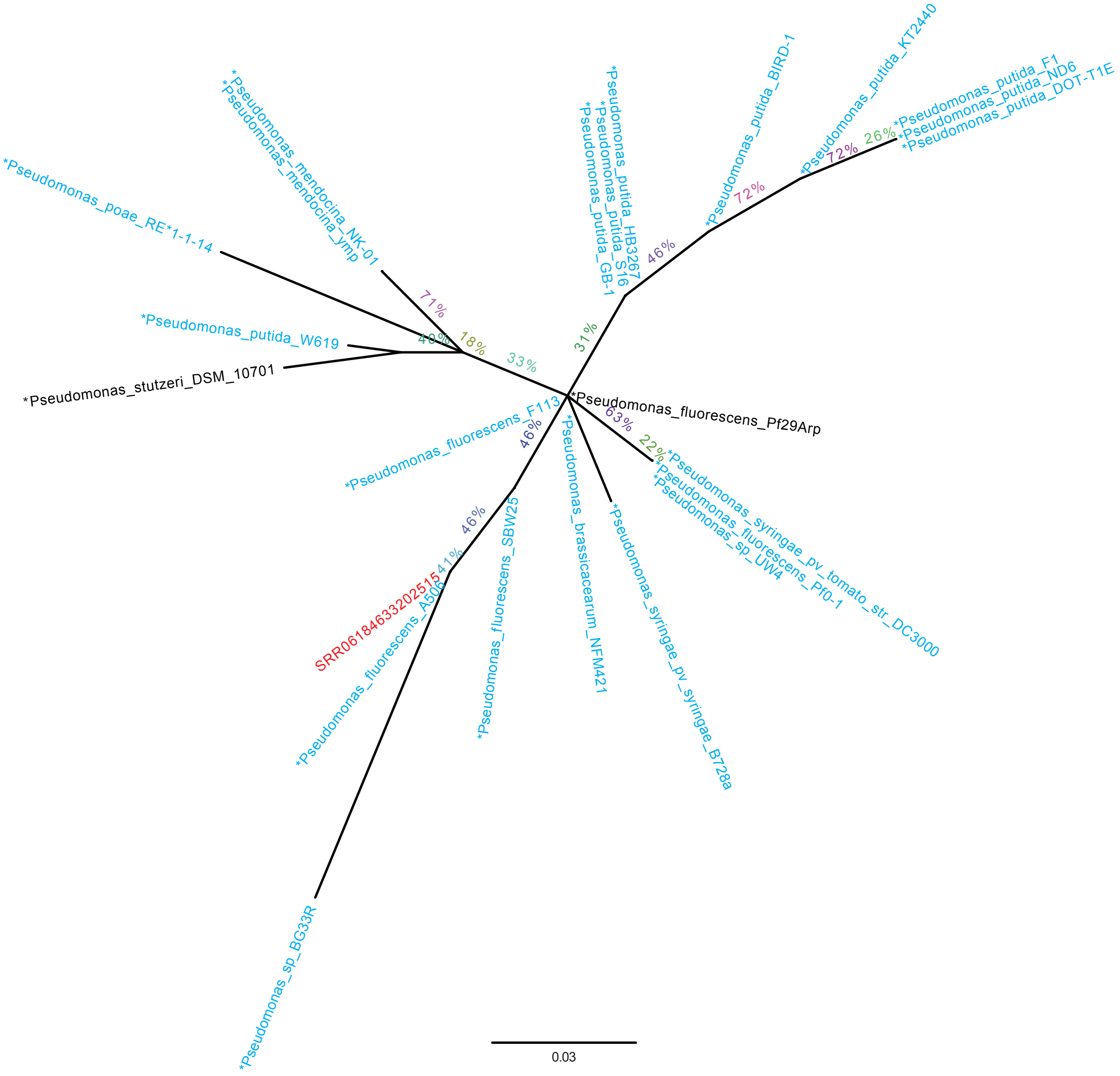

G

BWA LCA: Pseudomonadaceae
Blast LCA: *Pseudomonas*

a
*Pseudomonas_aeruginosa_23S
*Pseudomonas_fluorescens_rrnB
*Pseudomonas_fluorescens_rrnC
*Pseudomonas_syringae_pv__tomato_DC3000
*Pseudomonas_aeruginosa_DSM_50071T
*Pseudomonas_syringae_pv__phaseolicola
*Pseudomonas_syringae_pv__syringae
*Pseudomonas_aeruginosa_PAO1
*Pseudomonas_aeruginosa_UCBPP-PA14
*Pseudomonas_aeruginoas_PA7
*Pseudomonas_aeruginosa_LESB58
*Pseudomonas_fluorescens_SBW25
*Pseudomonas_aeruginosa_DB3
*Pseudomonas_brassicacearum
*Pseudomonas_aeruginosa_NCGM2_S1
*Pseudomonas_fluorescens_F113
*Pseudomonas_fluorescens_A506
Spodoptera_exigua_comp1146_c0_seq1 (moth)
*Pseudomonas_brassicacearum_NFM421
SRR203141.1048355
*Pseudomonas_syringae_pv__tomato
*Pseudomonas_poae_RE*1-1-14
*Pseudomonas_aeruginosa_M18
*Pseudomonas_aeruginosa_DK2
*Pseudomonas_fluorescens_Pf29Arp
*Pseudomonas_aeruginosa_B136-33

b
*Pseudomonas_perfectomarina_23S
*P_stutzeri_23S_rRNA_gene
*Pseudomonas_stutzeri
*Pseudomonas_stutzeri_A1501-1
*Pseudomonas_stutzeri_DSM_4166
*Pseudomonas_stutzeri_ATCC_17588
*Pseudomonas_stutzeri_CCUG_29243
*Pseudomonas_stutzeri_RCH2
*Pseudomonas_stutzeri_A1501

c
*Pseudomonas_putida_KT2440
Rubrobacter_radiotolerans_23S
*Pseudomonas_entomophila_L48
*Pseudomonas_putida_F1
*Pseudomonas_putida_W619
*Pseudomonas_putida_BIRD-1
*Pseudomonas_putida_S16
*Pseudomonas_putida_ND6
*Pseudomonas_putida_DOT-T1E
*Pseudomonas_putida_HB3267

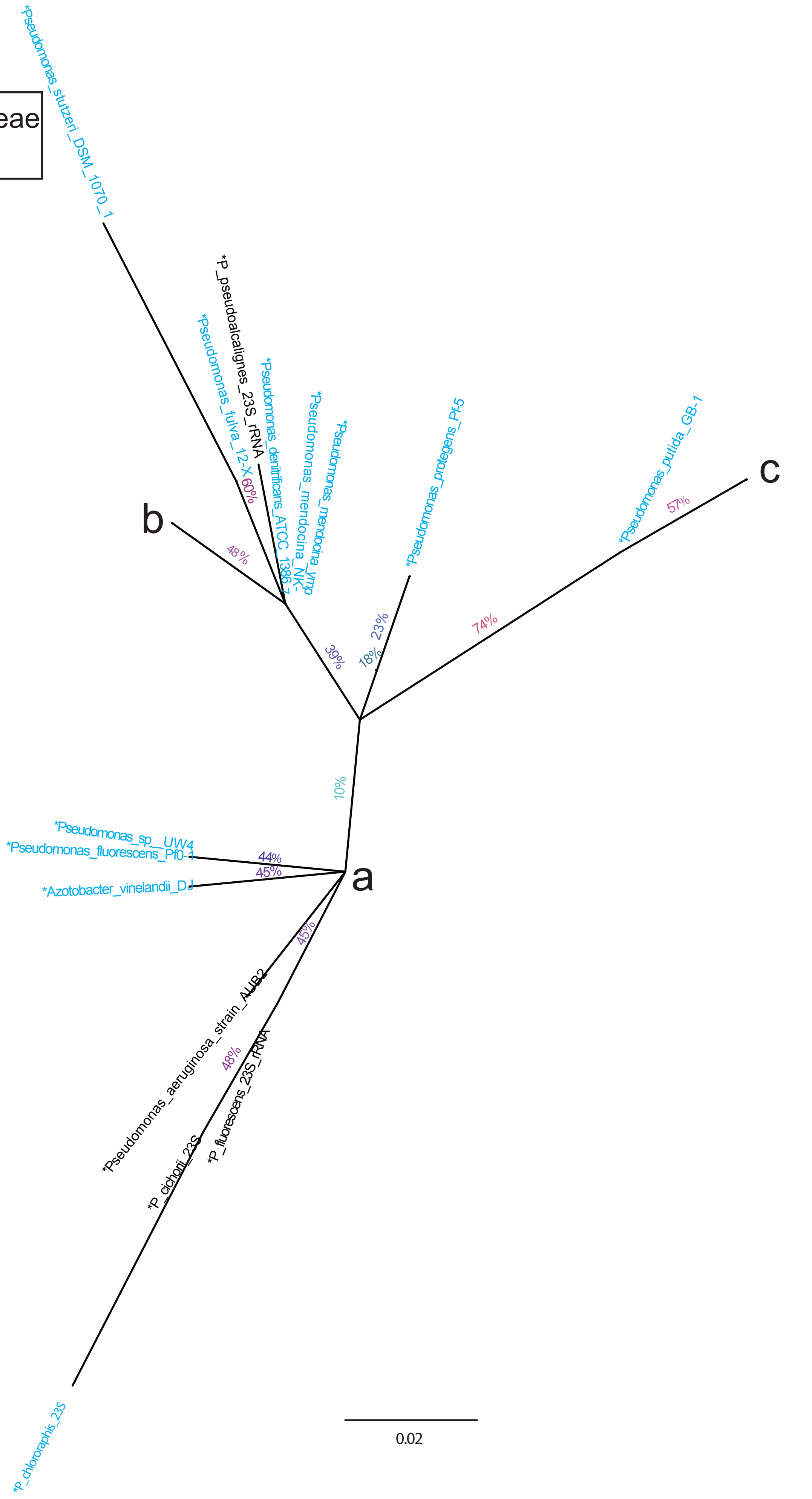

H

BWA LCA: Pseudomonadaceae
Blast LCA: Cellular Organism

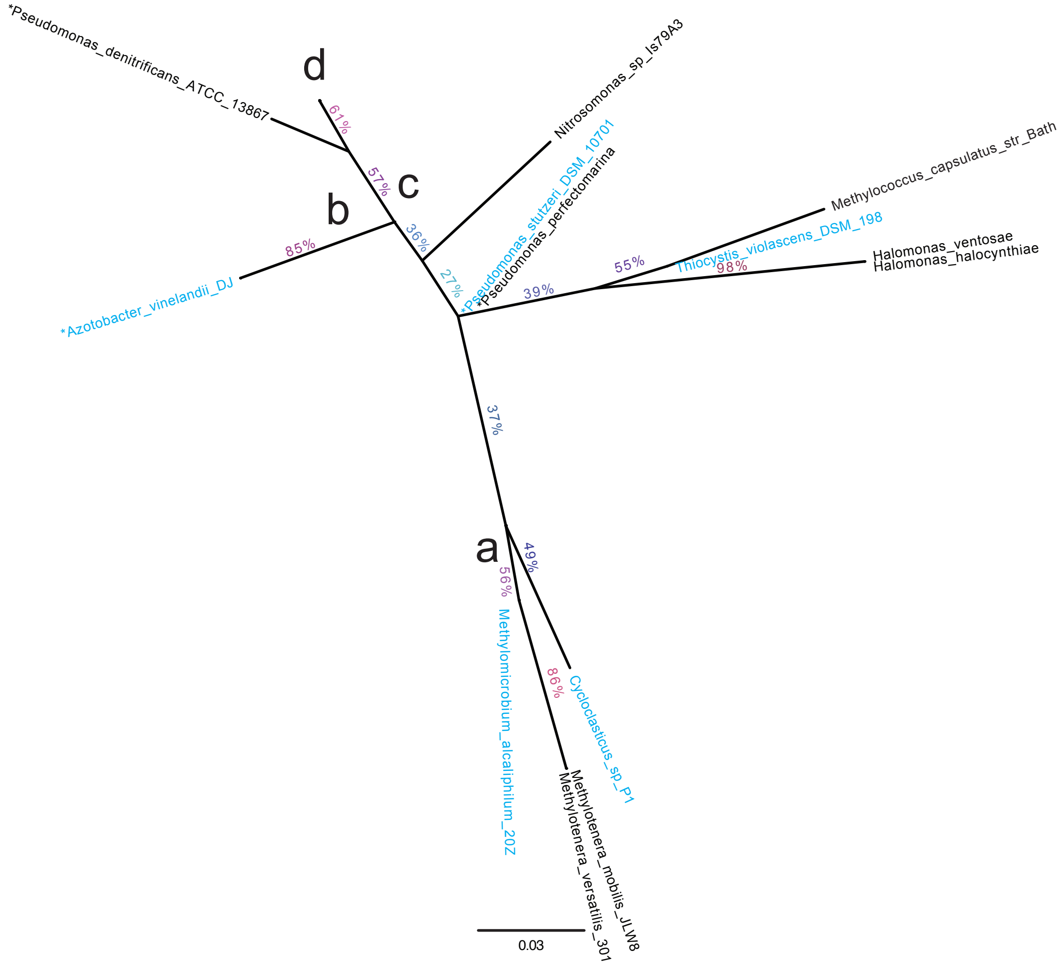

a
SRR061837.18908151
*Pseudomonas_brassicacearum_NFM421
*Pseudomonas_stutzeri_A1501
*Pseudomonas_protegens_Pf-5
*Pseudomonas_syringae_pv_phaseolicola_1448A
*Pseudomonas_syringae_pv_tomato_str_DC3000
*Pseudomonas_fluorescens_Pf29Arp
*Pseudomonas_poae_RE*1-1-14
*Pseudomonas_stutzeri_RCH2
*Pseudomonas_sp_UW4
*Pseudomonas_stutzeri_CCUG_29243
*Pseudomonas_stutzeri_23S
*Pseudomonas_fluorescens
*Pseudomonas_syringae_pv_syringae_B728a
*Pseudomonas_fluorescens_SBW25
*Pseudomonas_fluorescens_Pf0-1
Albugo_laibachii_Nc14 (oomycete)
*Pseudomonas_stutzeri_DSM_4166
Albugo_laibachii_Alem1 (oomycete)
*Pseudomonas_stutzeri_ATCC_17588
*Pseudomonas_fluorescens_F113
*Pseudomonas_fluorescens_A506

b
*Pseudomonas_entomophila_str_L48
*Pseudomonas_mendocina_NK-01
*Pseudomonas_mendocina_ymp
*Pseudomonas_fulva_12-X
*Pseudomonas_putida_F1
*Pseudomonas_putida_KT2440

c
*Pseudomonas_putida_HB3267
*Pseudomonas_putida_DOT-T1E
*Pseudomonas_putida_ND6
*Pseudomonas_putida_S16
*Pseudomonas_putida_BIRD-1
*Pseudomonas_putida_W619
*Pseudomonas_putida_GB-1

d
*Pseudomonas_aeruginosa
*Pseudomonas_aeruginosa_strain_ATCC_15014
*Pseudomonas_aeruginosa_UCBPP-PA14
*Pseudomonas_aeruginosa_PA7
*Pseudomonas_aeruginosa_LESB58
*Pseudomonas_aeruginosa_strain_DB3
*Pseudomonas_aeruginosa_strain_AUB2
*Pseudomonas_aeruginosa_M18
*Pseudomonas_aeruginosa_NCGM2S1
*Pseudomonas_aeruginosa_DK2
*Pseudomonas_aeruginosa_PAO1
*Pseudomonas_aeruginosa_B136-33

I

BWA LCA: Pseudomonadaceae
Blast LCA: Pseudomonadaceae

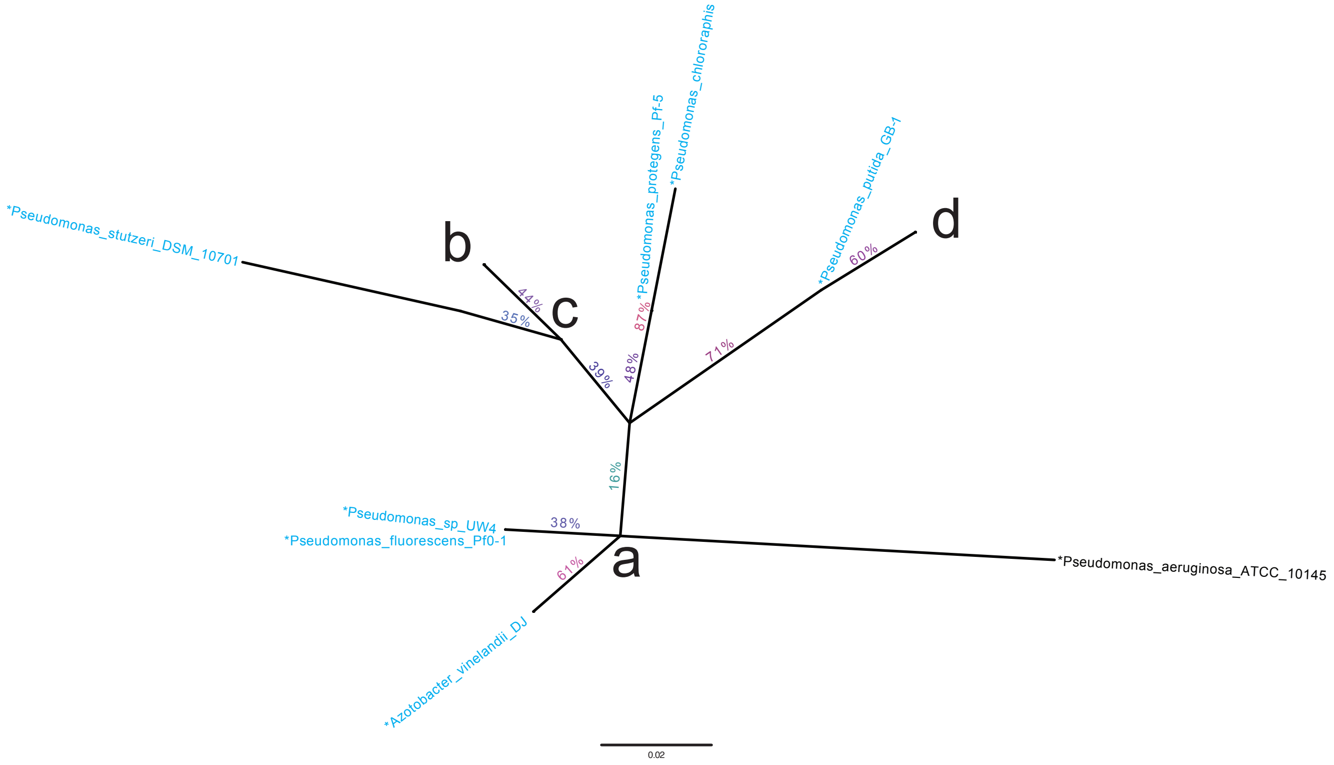

a
*Pseudomonas_aeruginosa_AUB2
*Pseudomonas_fluorescens
*Pseudomonas_aeruginosa
*Pseudomonas_aeruginosa_UCBPP-PA14
*Pseudomonas_aeruginosa_DB3
*Pseudomonas_fluorescens_A506
*Pseudomonas_aeruginosa_DK2
*Pseudomonas_fluorescens_F113
*Pseudomonas_fluorescens_SBW25
*Pseudomonas_syringae_pv_syringae_UMAF0158
*Pseudomonas_syringae_pv_syringae_B728a
*Pseudoimonas_cichori
*Pseudomonas_fluorescens
*Pseudomonas_poae_RE*1-1-14
SRR061897.14995429
*Pseudomonas_aeruginosa_B136-33
*Pseudomonas_brassicacearum_NFM421
*Pseudomonas_syringae_pv_phaseolicola_1448A
*Pseudomonas_syringae_pv_tomato_str_DC3000
*Pseudomonas_aeruginosa_PAO1

b
*Pseudomonas_fulva_12-X
*Pseudomonas_perfectomarina
*Pseudomonas_stutzeri
*Pseudomonas_stutzeri_DSM_4166
*Pseudomonas_stutzeri_ATCC_17588
*Pseudomonas_stutzeri_CCUG_29243
*Pseudomonas_stutzeri_RCH2
*Pseudomonas_stutzeri_A1501

c
*Pseudomonas_mendocina_NK-01
*Pseudomonas_mendocina_ymp
*Pseudomonas_pseudoalcaligenes
*Pseudomonas_denitrificans_ATCC_13867

d
Rubrobacter_radiotolerans
*Pseudomonas_entomophila_str_L48
*Pseudomonas_putida_W619
*Pseudomonas_putida_BIRD-1
*Pseudomonas_putida_S16
*Pseudomonas_putida_ND6
*Pseudomonas_putida_DOT-T1E
*Pseudomonas_putida_HB3267
*Pseudomonas_putida_KT2440
*Pseudomonas_putida_F1

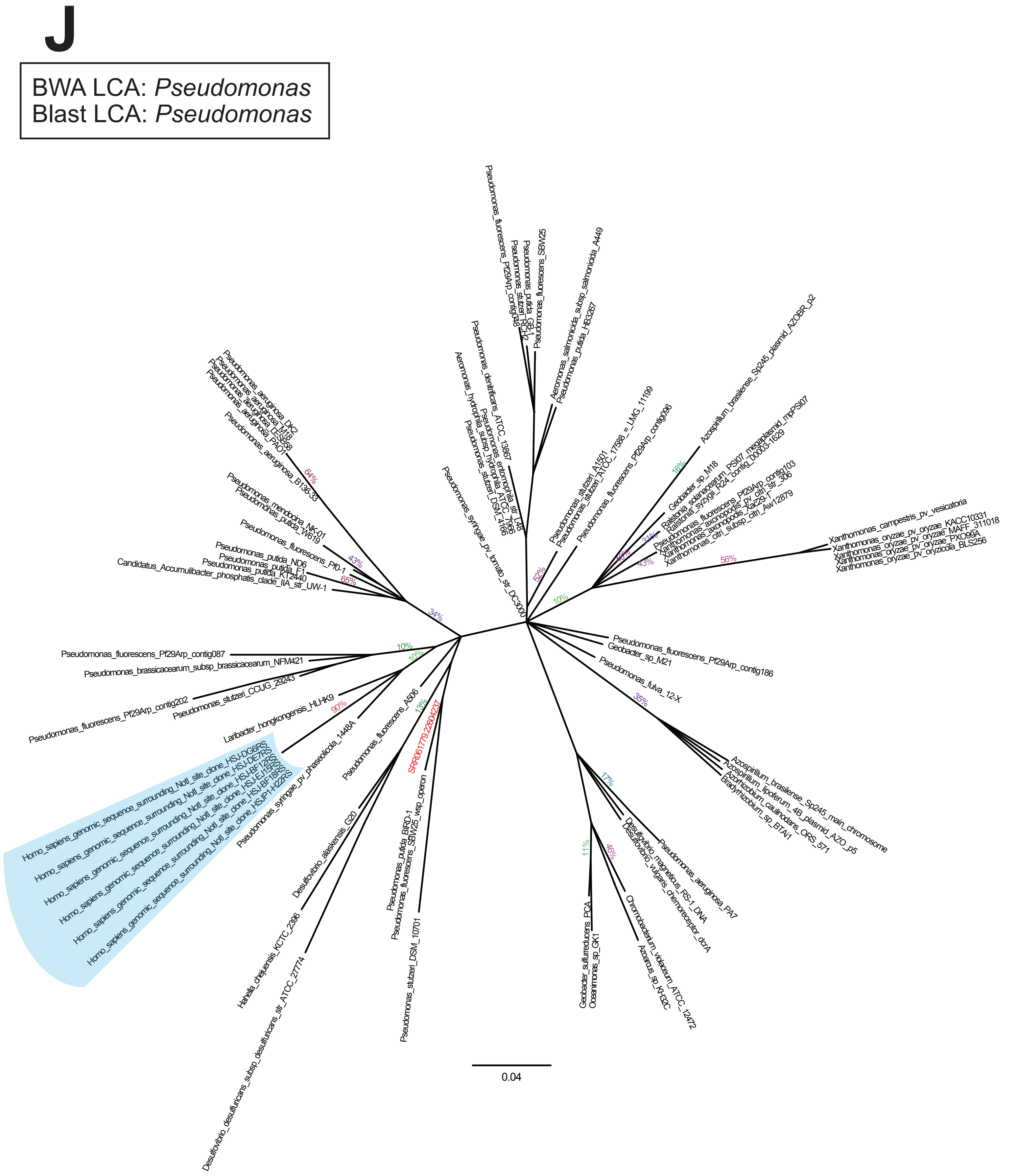
J
BWA LCA: *Pseudomonas*
Blast LCA: *Pseudomonas*
Pseudomonas_fluorescens_SBW25
Pseudomonas_putida_GB-1
Pseudomonas_stutzeri_RCH2
Pseudomonas_fluorescens_Pf29Arp_contig048
Aeromonas_salmonicida_subsp_salmonicida_A449
Pseudomonas_putida_HB3267
Pseudomonas_denitrificans_ATCC_13867
Aeromonas_hydrophila_subsp_hydrophila_ATCC_7966
Pseudomonas_entomophila_str_L48
Pseudomonas_stutzeri_DSM_4166
Pseudomonas_syringae_pv_tomato_str_DC3000
Pseudomonas_stutzeri_A1501
Pseudomonas_stutzeri_ATCC_17588_=_LMG_11199
Pseudomonas_fluorescens_Pf29Arp_contig096
Azospirillum_brasilense_Sp245_plasmid_AZOBR_p2
Geobacter_sp_M18
Ralstonia_solanacearum_PSI07_megaplasmid_mpPSI07
Ralstonia_syzygii_R24_contig_0003-1629
Pseudomonas_fluorescens_Pf29Arp_contig103
Xanthomonas_axonopodis_pv_citri_str_306
Xanthomonas_axonopodis_Xac29-1
Xanthomonas_citri_subsp_citri_Aw12879
Xanthomonas_campestris_pv_vesicatoria
Xanthomonas_oryzae_pv_oryzae_KACC10331
Xanthomonas_oryzae_pv_oryzae_MAFF_311018
Xanthomonas_oryzae_pv_oryzae_PXO99A
Xanthomonas_oryzae_pv_oryzicola_BLS256
Pseudomonas_aeruginosa_DK2
Pseudomonas_aeruginosa_M18
Pseudomonas_aeruginosa_LESB58
Pseudomonas_aeruginosa_PAO1
Pseudomonas_aeruginosa_B136-33
Pseudomonas_mendocina_NK-01
Pseudomonas_putida_W619
Pseudomonas_fluorescens_Pf0-1
Pseudomonas_putida_ND6
Pseudomonas_putida_F1
Pseudomonas_putida_KT2440
Candidatus_Accumulibacter_phosphatis_clade_IIA_str_UW-1
Pseudomonas_fluorescens_Pf29Arp_contig087
Pseudomonas_brassicacearum_subsp_brassicacearum_NFM421
Pseudomonas_fluorescens_Pf29Arp_contig202
Pseudomonas_stutzeri_CCUG_29243
Laribacter_hongkongensis_HLHK9
Pseudomonas_syringae_pv_phaseolicola_1448A
Pseudomonas_fluorescens_A506
SRR061779.2260423?
Pseudomonas_putida_BIRD-1
Pseudomonas_fluorescens_SBW25_wsp_operon
Pseudomonas_stutzeri_DSM_10701
Homo_sapiens_genomic_sequence_surrounding_NotI_site_clone_HSJ-DG6RS
Homo_sapiens_genomic_sequence_surrounding_NotI_site_clone_HSJ-DE7RS
Homo_sapiens_genomic_sequence_surrounding_NotI_site_clone_HSJ-BF12RS
Homo_sapiens_genomic_sequence_surrounding_NotI_site_clone_HSJ-EJ15RS
Homo_sapiens_genomic_sequence_surrounding_NotI_site_clone_HSJ-BF18RS
Homo_sapiens_genomic_sequence_surrounding_NotI_site_clone_HSJP1-H22RS
Desulfovibrio_alaskensis_G20
Hahella_chejuensis_KCTC_2396
Desulfovibrio_desulfuricans_subsp_desulfuricans_str_ATCC_27774
Pseudomonas_fluorescens_Pf29Arp_contig186
Geobacter_sp_M21
Pseudomonas_fulva_12-X
Azospirillum_brasilense_Sp245_main_chromosome
Azospirillum_lipoferum_4B_plasmid_AZO_p5
Azorhizobium_caulinodans_ORS_571
Bradyrhizobium_sp_BTAi1
Pseudomonas_aeruginosa_PA7
Desulfovibrio_magneticus_RS-1_DNA
Desulfovibrio_vulgaris_chemoreceptor_dcrA
Geobacter_sulfurreducens_PCA
Oceanimonas_sp_GK1
Chromobacterium_violaceum_ATCC_12472
Azoarcus_sp_KH32C
64%
43%
65%
34%
10%
10%
90%
13%
52%
10%
16%
34%
46%
43%
56%
35%
17%
11%
46%
0.04
