# Supplementary material for: Bacteria-Human Somatic Cell Lateral Gene Transfer Is Enriched in Cancer Samples
Source: PLoS Comput Biol. 2013 Jun 20;9(6):e1003107. doi: 10.1371/journal.pcbi.1003107 (PMC3688693; doi:10.1371/journal.pcbi.1003107)
Supplement: Table S5 — Abnormal insert sizes for Illumina libraries. The percentage of reads with abnormal insert sizes for experimental and control samples is listed for the data presented in Figure S9. (PDF) [file pcbi.1003107.s015.pdf]

**Table S3. Percentage of reads with abnormal insert sizes for experimental and control samples**

| Panel in Figure S7 | Number of pairs with >600 bp insert | Percentage of pairs with > 600 bp insert |
|---|---|---|
| A | 150 | 0.055 |
| B | 1578 | 0.60 |
| C | 1551 | 0.57 |
| D | 273 | 0.19 |
| E | 263 | 0.65 |
| F | 191 | 0.80 |
| G | 297 | 0.73 |
| H | 12143 | 0.87 |
| I | 29430 | 0.23 |
| J | 26902 | 0.22 |
| K | 31854 | 0.26 |
| L | 34639 | 0.29 |
| M | 112579 | 0.97 |
| N | 101588 | 0.94 |
| O | 117153 | 1.1 |
| P | 109138 | 1.0 |
